# Supplementary material for: Heterogeneity of neuroendocrine transcriptional states in metastatic small cell lung cancers and patient-derived models
Source: Nat Commun. 2022 Apr 19;13:2023. doi: 10.1038/s41467-022-29517-9 (PMC9018864; doi:10.1038/s41467-022-29517-9)
Supplement: Supplementary file 1 — Supplementary Information [file 41467_2022_29517_MOESM1_ESM.pdf]

Supplementary Information

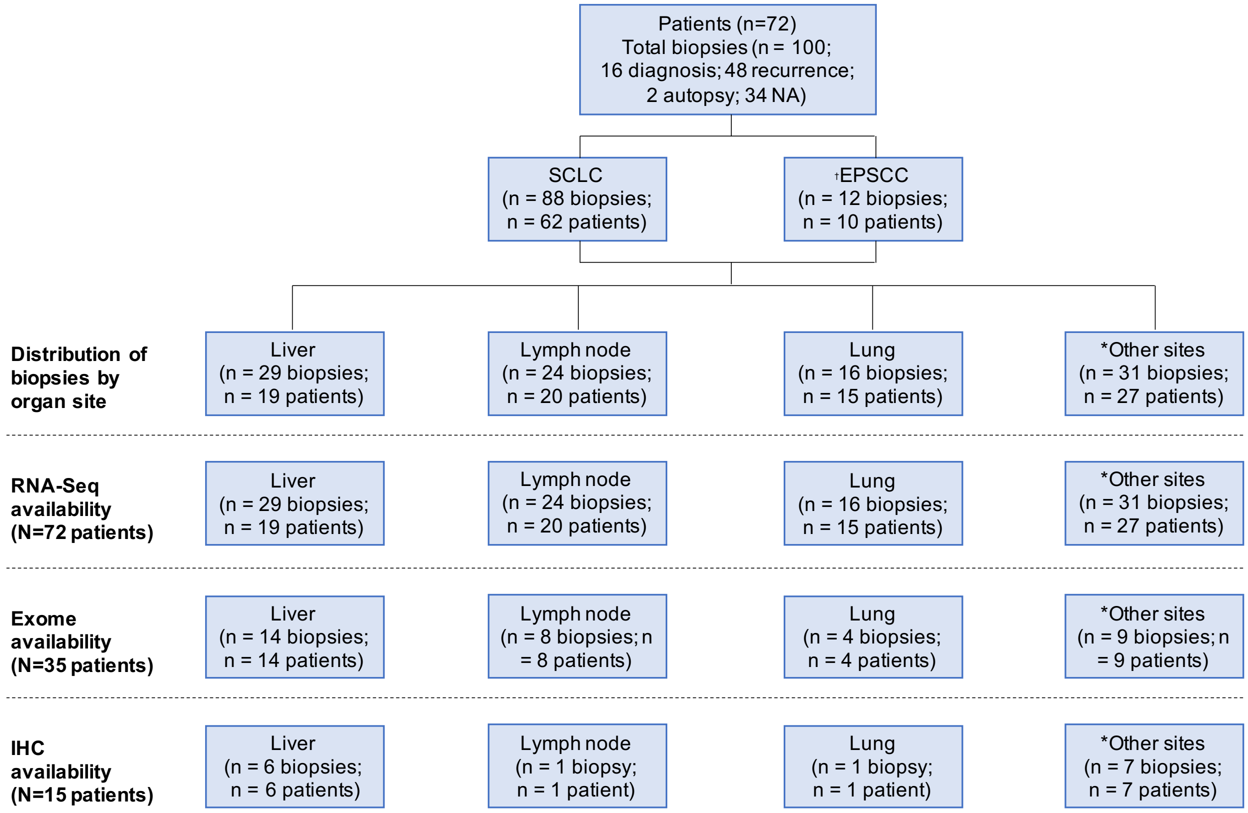

†Including EGFRmut SCLC

*Other sites (n=biopsies):
Adrenal mass, Bladder, Brain, Chest wall, Mediastinal mass, Pelvis mass, Pleura fluid and soft tissue, Prostate, Subcarinal mass, Uterine cervix

Supplementary Figure 1

Biopsy characteristics. Abbreviations: SCLC: small cell lung cancer; EPSCC: extrapulmonary small cell cancer.

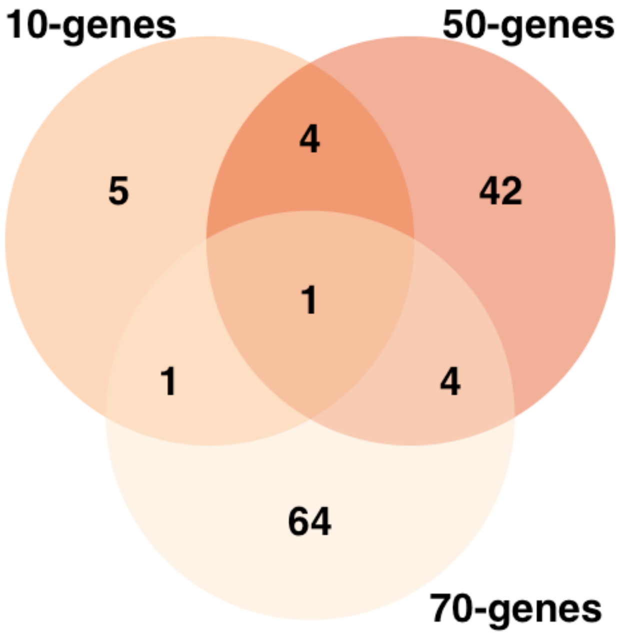

Supplementary Figure 2
The Venn diagram shows the overlaps and differences between gene lists.

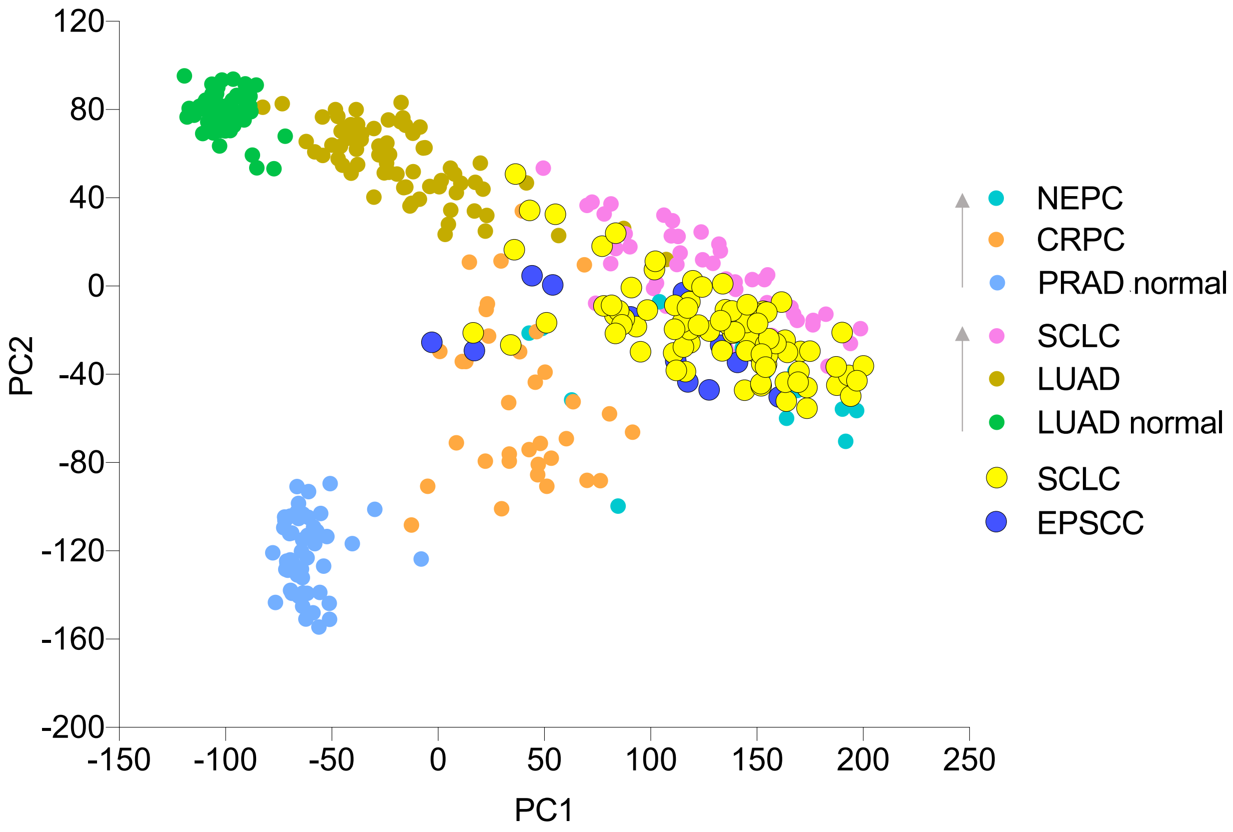

Supplementary Figure 3
Projection of the 100 SCNC tumors – SCLC (yellow) and EPSCC (blue) – onto the PCA developed by Balanis et al.[8], to evaluate the degree of neuroendocrine differentiation (trajectory indicated by arrows). Abbreviations: SCLC: small cell lung cancer; EPSCC: extrapulmonary small cell cancer; NEPC: neuroendocrine prostate cancer; CRPC: castration-resistant prostate cancer; PRAD: prostate adenocarcinoma; LUAD: lung adenocarcinoma; PCA: principal component analysis.

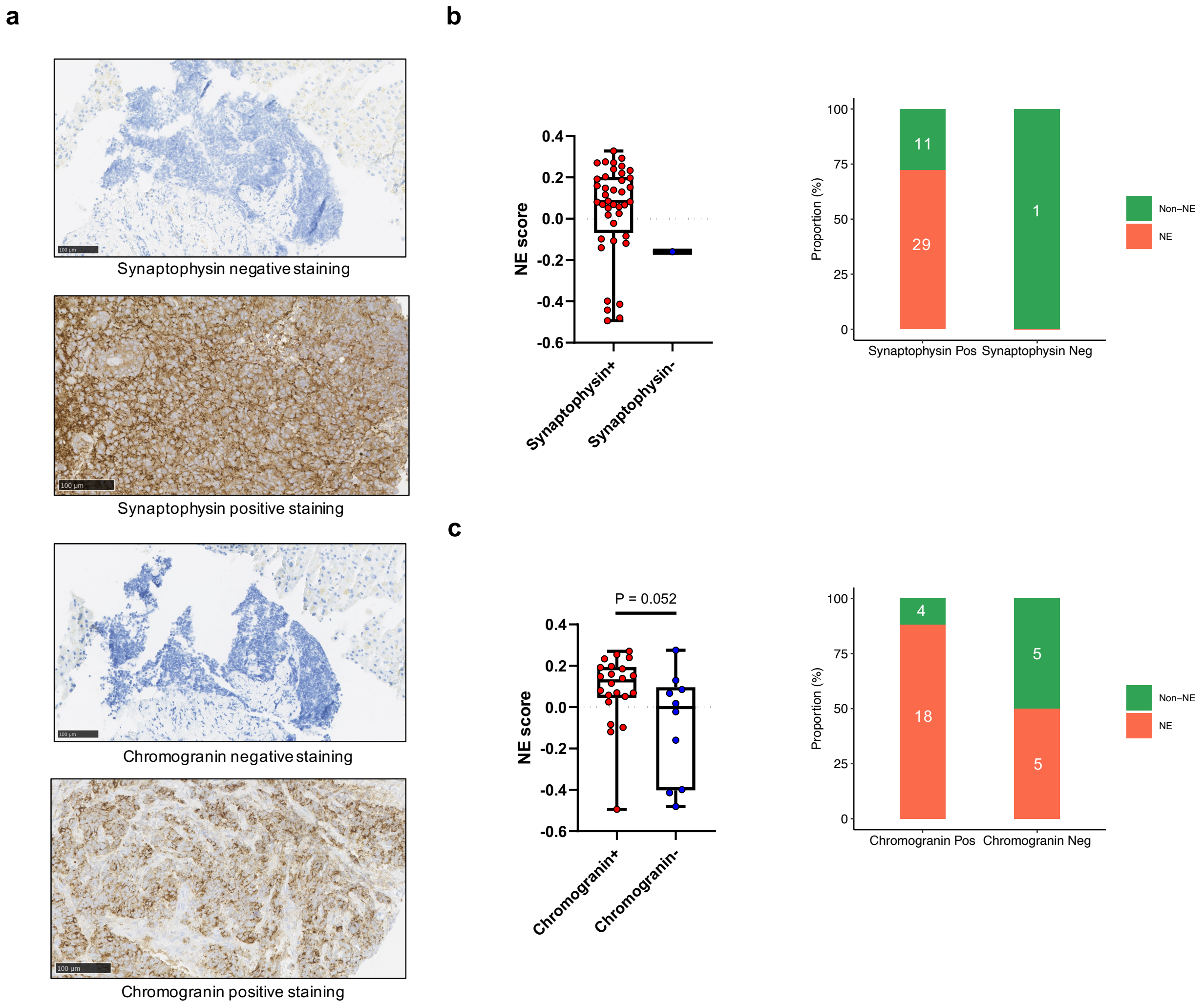

Supplementary Figure 4

(a) Representative IHC images of synaptophysin and chromogranin (observations were repeated independently 2 times). (b-c) NE score distribution based on (b) synaptophysin (n=41 tumors) and (c) chromogranin (n=32 tumors) expression. The proportion of NE and non-NE subtypes in IHC stained samples is indicated on the right panels. All box plots indicate the inter-quartile range (IQR), the middle line corresponds to the median, and the upper and lower whiskers represent observations within 1.5*IQR (Q3 + 1.5*IQR or Q1 − 1.5*IQR). Two-tailed Mann-Whitney U-test. Abbreviations: IHC: immunohistochemistry; NE: neuroendocrine differentiation.

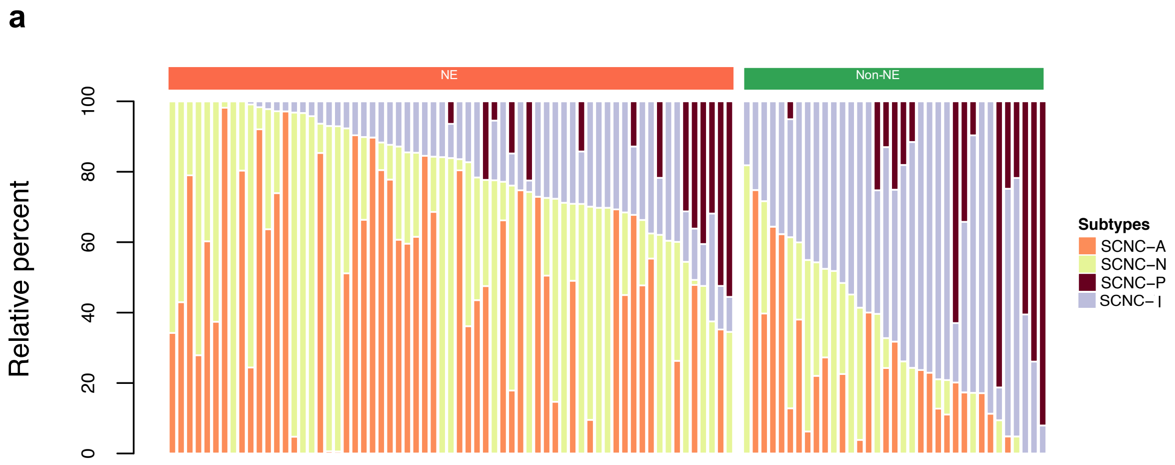

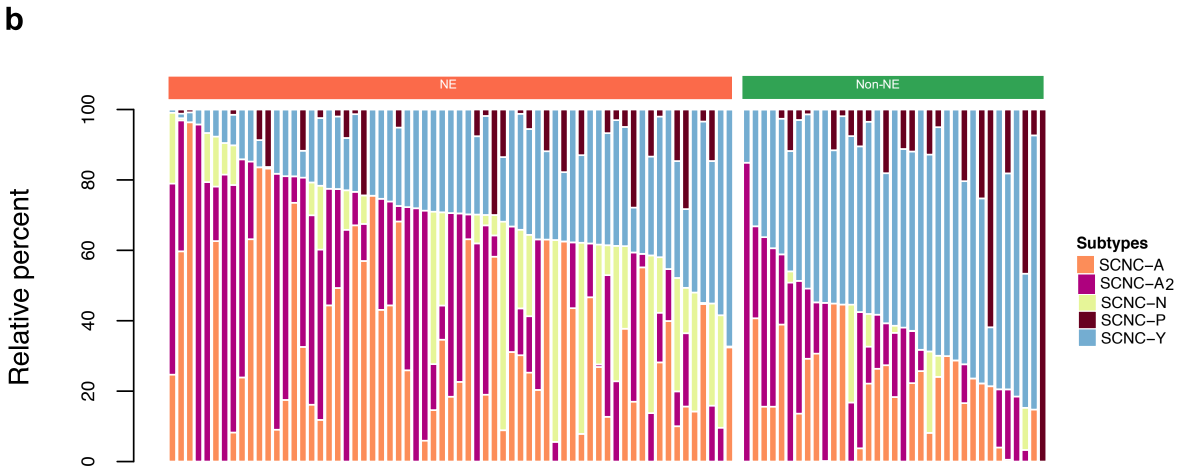

Supplementary Figure 5
CIBERSORT analysis[25] of subtype-specific gene matrices published by (a) Ireland et al[31] and (b) Groves et al[26] grouped by NE subtype. Abbreviation: SCNC: small cell neuroendocrine cancers.

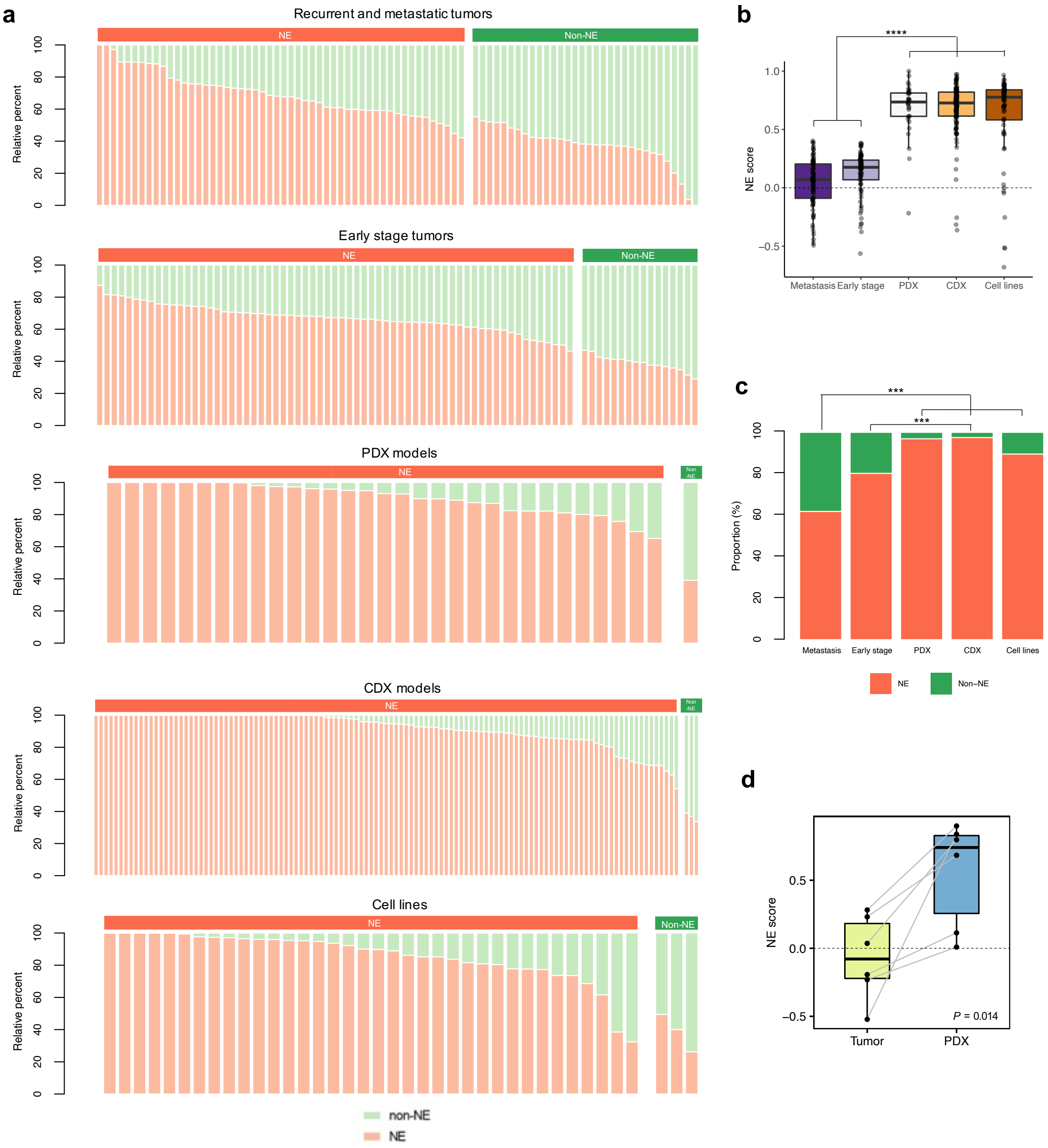
a
Recurrent and metastatic tumors
NE
Non-NE
Relative percent
Early stage tumors
PDX models
CDX models
Cell lines
non-NE
NE
b
NE score
****
Metastasis
Early stage
PDX
CDX
Cell lines
c
***
***
Proportion (%)
NE
Non–NE
d
NE score
P = 0.014
Tumor
PDX

Supplementary Figure 6

(a) CIBERSORT analysis[25] of the 50-gene signature in 84 recurrent and metastatic tumors of this cohort and previously described cohorts of 81early-stage tumors[30], 32 PDX, 120 CDX[22,28] models, and 39 immortalized cell lines[33,34] grouped by NE subtype. (b) NE score distribution across metastatic and early-stage tumors, and patient-derived models. Kruskal-Wallis test followed by Dunn's multiple comparisons test with BH correction, ****$P < 0.0001$ (ranging from P=7.50e-30 to 7.62e-12). (c) Proportion of NE and non-NE tumors based on NE score in metastatic and early-stage tumors, PDX, CDX models, and immortalized cell lines. Fisher's exact test followed by BH multiple comparison test, ***$P < 0.001$. (d) NE score distribution across 6 PDX and corresponding donor patient tumors. Paired t-test, $P = 0.014$. All tests are two-tailed. All box plots indicate the inter-quartile range (IQR), the middle line corresponds to the median, and the upper and lower whiskers represent observations within 1.5*IQR (Q3 + 1.5*IQR or Q1 − 1.5*IQR). Abbreviations: NE: neuroendocrine differentiation; PDX: patient-delivered xenografts; CDX: CTC-derived xenografts.

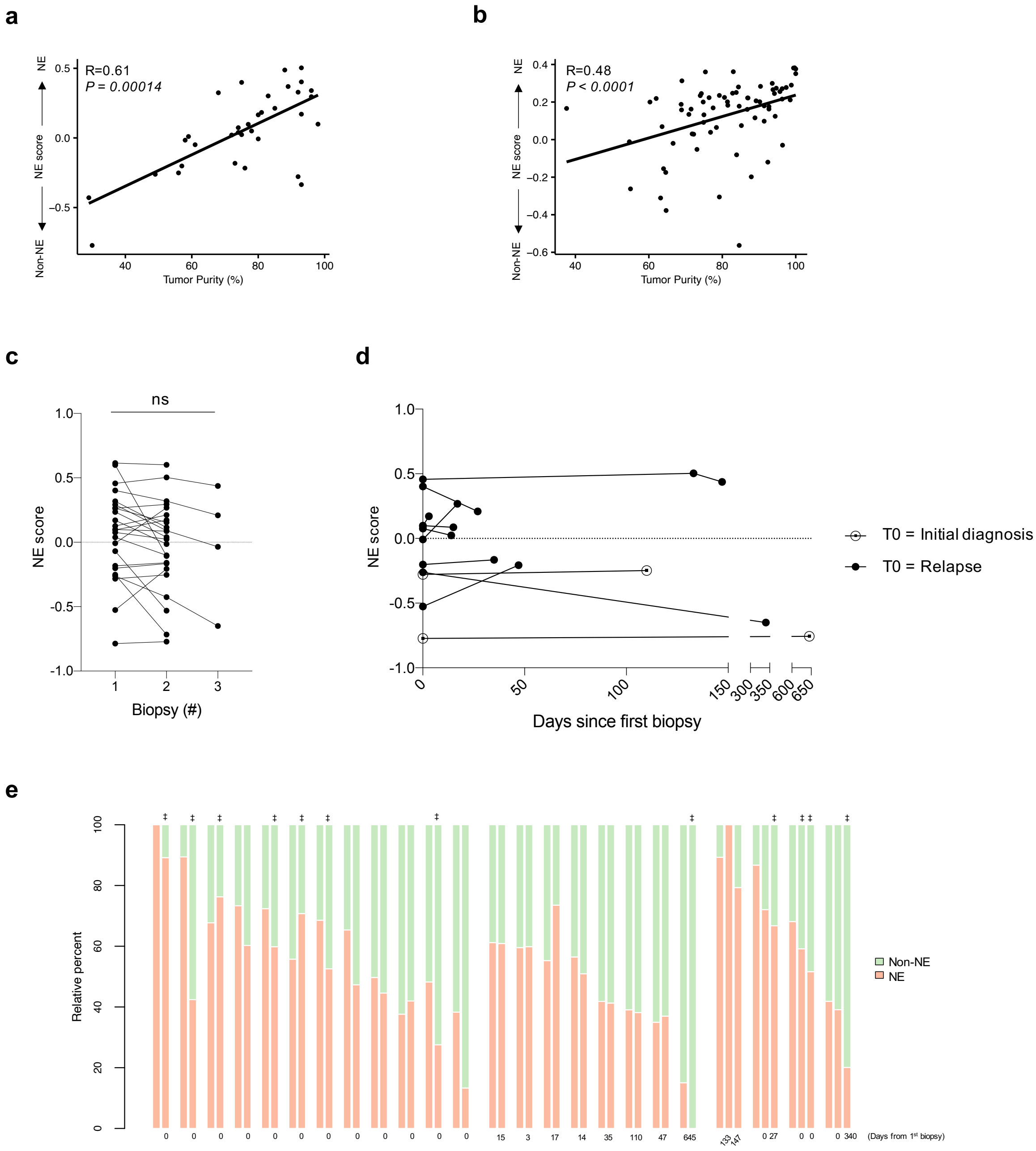

Supplementary Figure 7

Spearman correlation between tumor purity and neuroendocrine score in (a) 34 metastatic tumors of this cohort and (b) a previously described cohort of 71 early-stage tumors[30]. Spearman R values and *P*-values are indicated. (c) NE scores for multiple samples obtained from the same patient (including sequential biopsies, multiple samples collected at different sites on same day and serial tumor sections). (d) NE scores for sequential biopsies over time. (e) CIBERSORT analysis[25] of the 50-gene signature in multiple samples obtained from the same patient. The number of days from the first biopsy are displayed (0 = serial tumor section or different biopsies obtained on the same day). ‡ indicates different biopsy sites between samples. Fisher's exact test for each patient, ns. All tests are two-tailed. Abbreviation: NE: neuroendocrine differentiation.

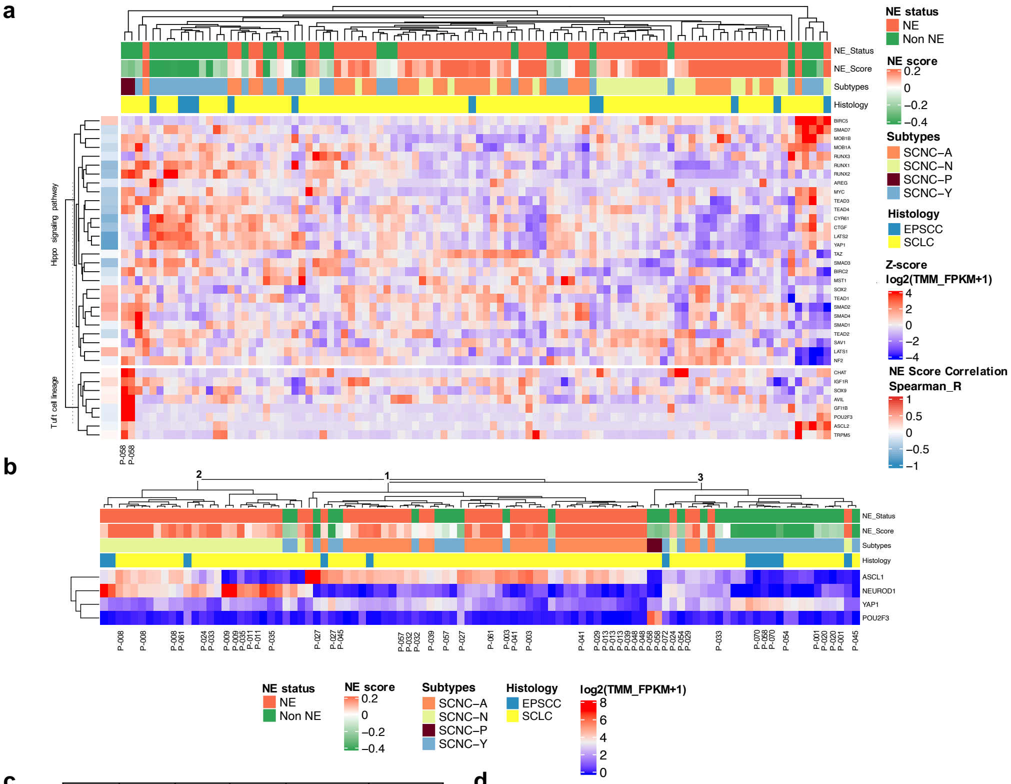

c

Patient_id	Biopsy 1	Biopsy 2	Biopsy 3	Biopsy collection	Discrepancy
P-001	Y	Y		Serial section	No
P-003	A	A > Y		Serial section	No
P-008	N	N	N	Combination	No
P-009	N	N		Sequential	No
P-010	Y	Y > A		Sequential	No
P-011	N	N		Sequential	No
P-013	A	A	A > Y	Sequential	No
P-020	Y	Y		Sequential	No
P-024	N	Y > N		Sequential	Yes
P-027	A > Y	A > Y	A > Y	Combination	No
P-029	A	Y > A		Multiple biopsies	Yes
P-032	A	A	A > Y	Multiple biopsies	No
P-033	N	Y		Multiple biopsies	Yes
P-035	N	N		Multiple biopsies	No
P-039	A	A		Multiple biopsies	No
P-041	A	A		Multiple biopsies	No
P-045	A >Y	Y		Multiple biopsies	Yes
P-048	A	A		Multiple biopsies	No
P-054	Y	Y		Sequential	No
P-057	A	A > Y		Serial section	No
P-058	P	P		Sequential	No
P-061	A	N		Serial section	Yes
P-070	Y	Y		Sequential	No
P-072	Y	Y		Serial section	No

79% agreement (19/24)

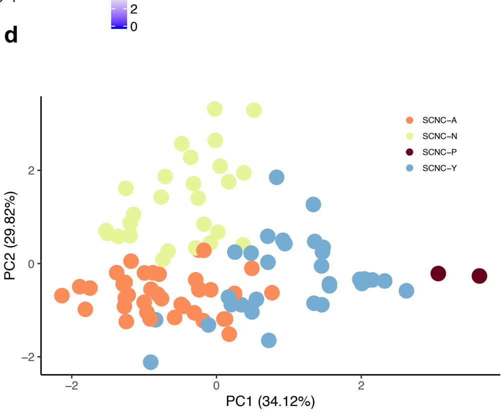

Supplementary Figure 8

(a) Heatmap generated by unsupervised hierarchal clustering of the tuft cell marker genes[37] and the Hippo signaling pathway genes[40,41]. NE scores, NE status, molecular subtypes and histology are indicated above the heatmap. (b) Heatmap generated by unsupervised hierarchal clustering of the four transcription factors in 100 tumors. Paired are indicated below the heatmap. (c) Table summarizing the molecular subtype classification for the multiple samples obtained for each patient. Combination refers to sequential biopsies and serial sections. Multiple biopsies indicate 2 or 3 separate sites taken on same day. (d) Supervised PCA using the expression of the four transcription factors. Each dot represents a tumor colored by molecular subtype. Abbreviations: SCNC: small cell neuroendocrine cancers; NE: neuroendocrine differentiation; SCLC: small cell lung cancer; EPSCC: extrapulmonary small cell cancer; TMM: Trimmed Mean of M-values; FPKM: Fragments Per Kilobase of Exon Per Million Fragments Mapped.

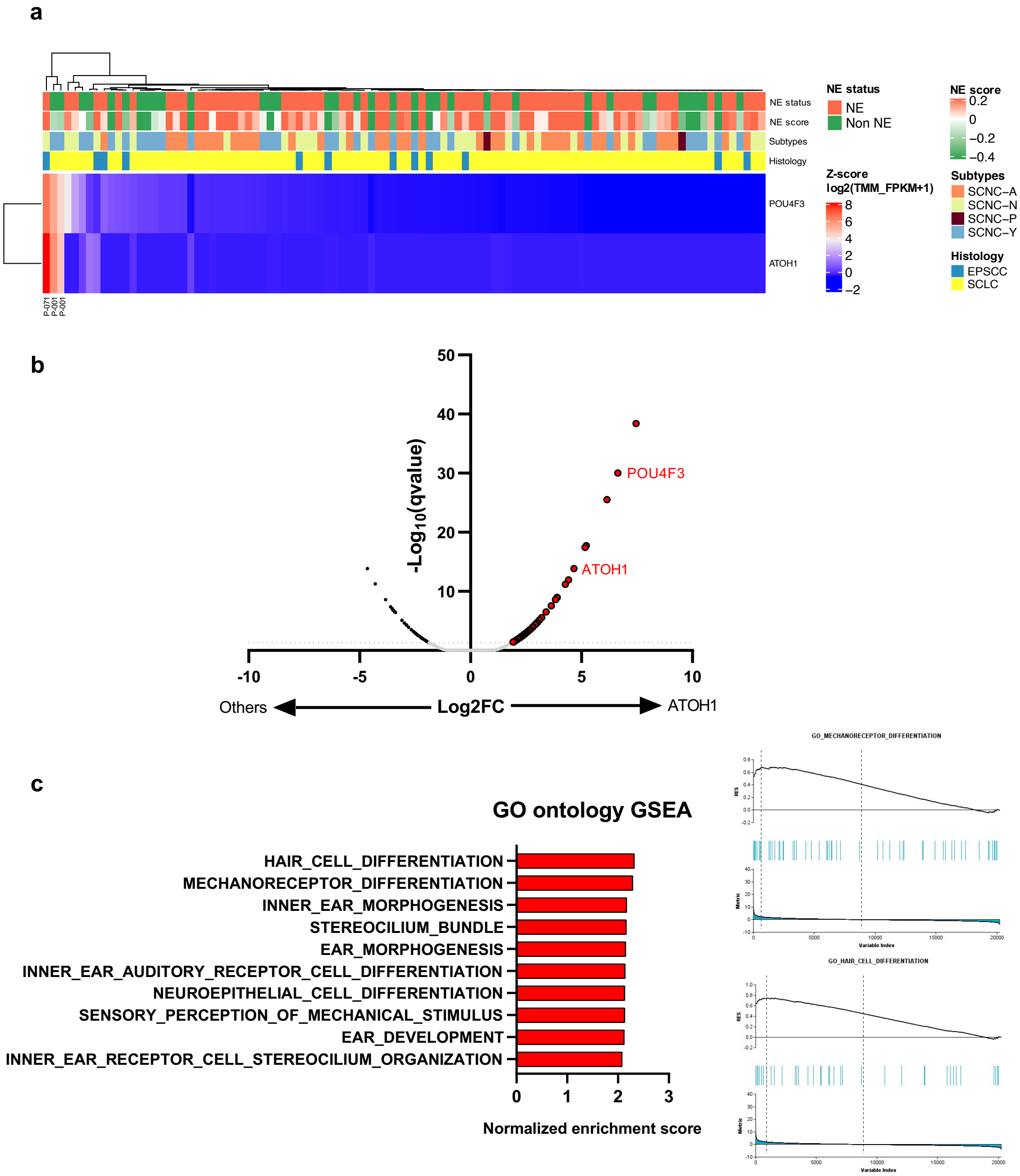

Supplementary Figure 9

(a) Heatmap generated by unsupervised hierarchal clustering of *ATOH1* and *POU4F3* gene expression in 100 tumors. (b) Volcano plot of the most differentially regulated genes between tumors expressing high *ATOH1* and *POU4F3*, and other tumors. (c) GSEA of Gene Ontology pathways in tumors expressing high *ATOH1* compared with other tumors. The top 10 gene sets with the highest normalized enrichment scores are shown. Abbreviations: SCNC: small cell neuroendocrine cancers; NE: neuroendocrine differentiation; SCLC: small cell lung cancer; EPSCC: extrapulmonary small cell cancer; GSEA: gene set enrichment analysis; TMM: Trimmed Mean of M-values; FPKM: Fragments Per Kilobase of Exon Per Million Fragments Mapped.

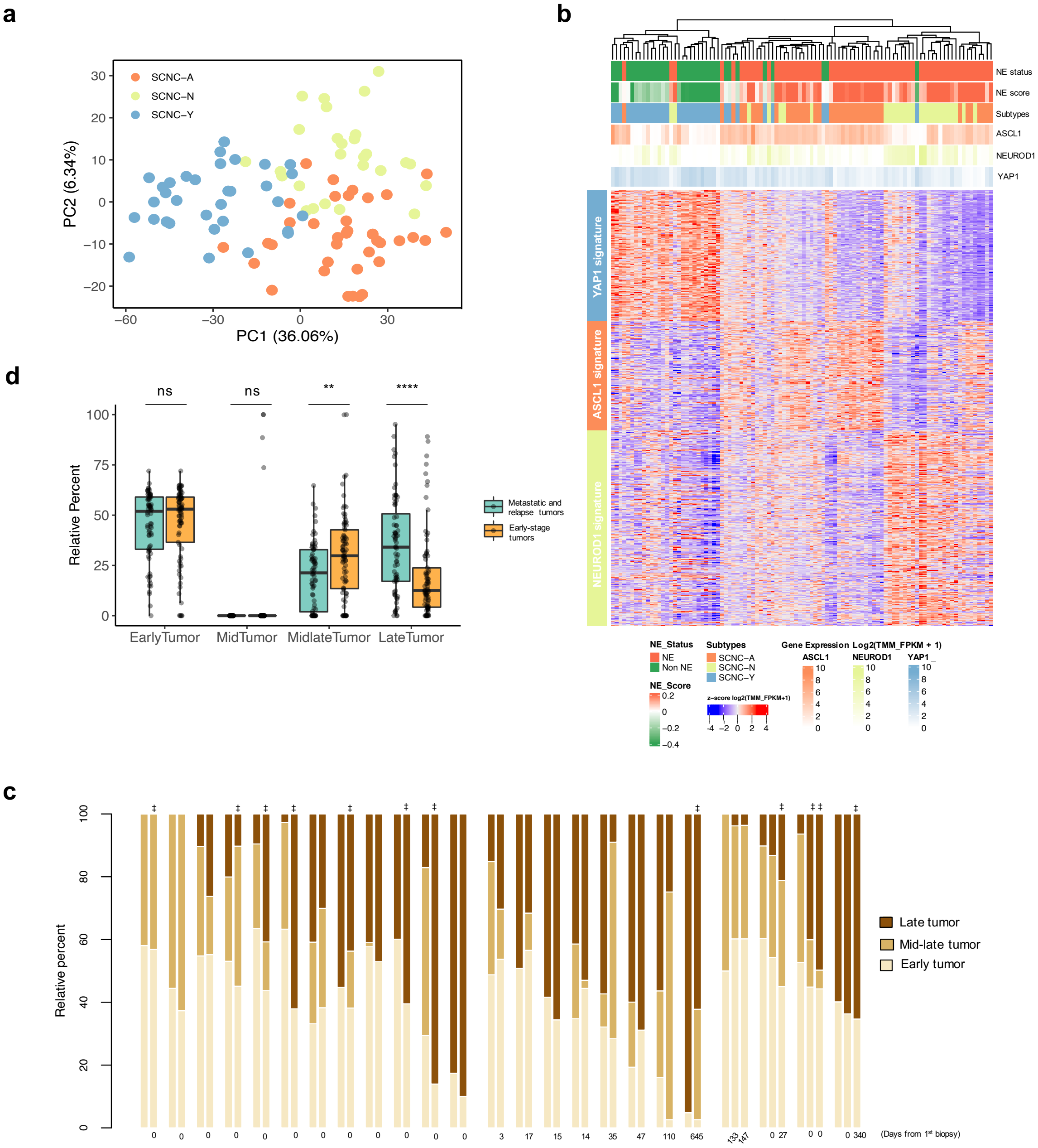
a
b
SCNC−A
SCNC−N
SCNC−Y
PC2 (6.34%)
PC1 (36.06%)
NE status
NE score
Subtypes
ASCL1
NEUROD1
YAP1
YAP1 signature
ASCL1 signature
NEUROD1 signature
NE_Status
NE
Non NE
NE_Score
Subtypes
Gene Expression Log2(TMM_FPKM + 1)
z-score log2(TMM_FPKM+1)
d
ns
ns
**
****
Relative Percent
EarlyTumor
MidTumor
MidlateTumor
LateTumor
Metastatic and relapse tumors
Early-stage tumors
c
Relative percent
Late tumor
Mid-late tumor
Early tumor
(Days from 1st biopsy)

Supplementary Figure 10
(a) Supervised PCA using the expression of the top 2000 differentially expressed genes across SCNC-A, -N, and -Y tumors. Each dot represents a patient colored by the SCNC subtype. (b) Heatmap clustered with Pearson's correlation and average linkage of the three subtype-specific gene signatures. (c) CIBERSORT analysis[25] of the gene signatures derived at different time points of MYC-driven tumor transition toward a non-NE phenotype[31], in multiple samples obtained from the same patient. The number of days from the first biopsy are displayed (0 = serial tumor section or different biopsies obtained on the same day). ‡ indicates different biopsy sites between samples. Fisher's exact test for each patient, ns. (d) Box plot of the relative proportion of early, mid, mid/late and late tumor phenotypes[31] in 72 metastatic and 81 early-stage tumors[30]. Two-tailed Mann-Whitney U-test, ****P = 1.39e-05, **P = 0.0032, ns, not significant. Box plots indicate the inter-quartile range (IQR), the middle line corresponds to the median, and the upper and lower whiskers represent observations within 1.5*IQR (Q3 + 1.5*IQR or Q1 − 1.5*IQR). Abbreviations: SCNC: small cell neuroendocrine cancers; NE: neuroendocrine differentiation; TMM: Trimmed Mean of M-values; FPKM: Fragments Per Kilobase of Exon Per Million Fragments Mapped.

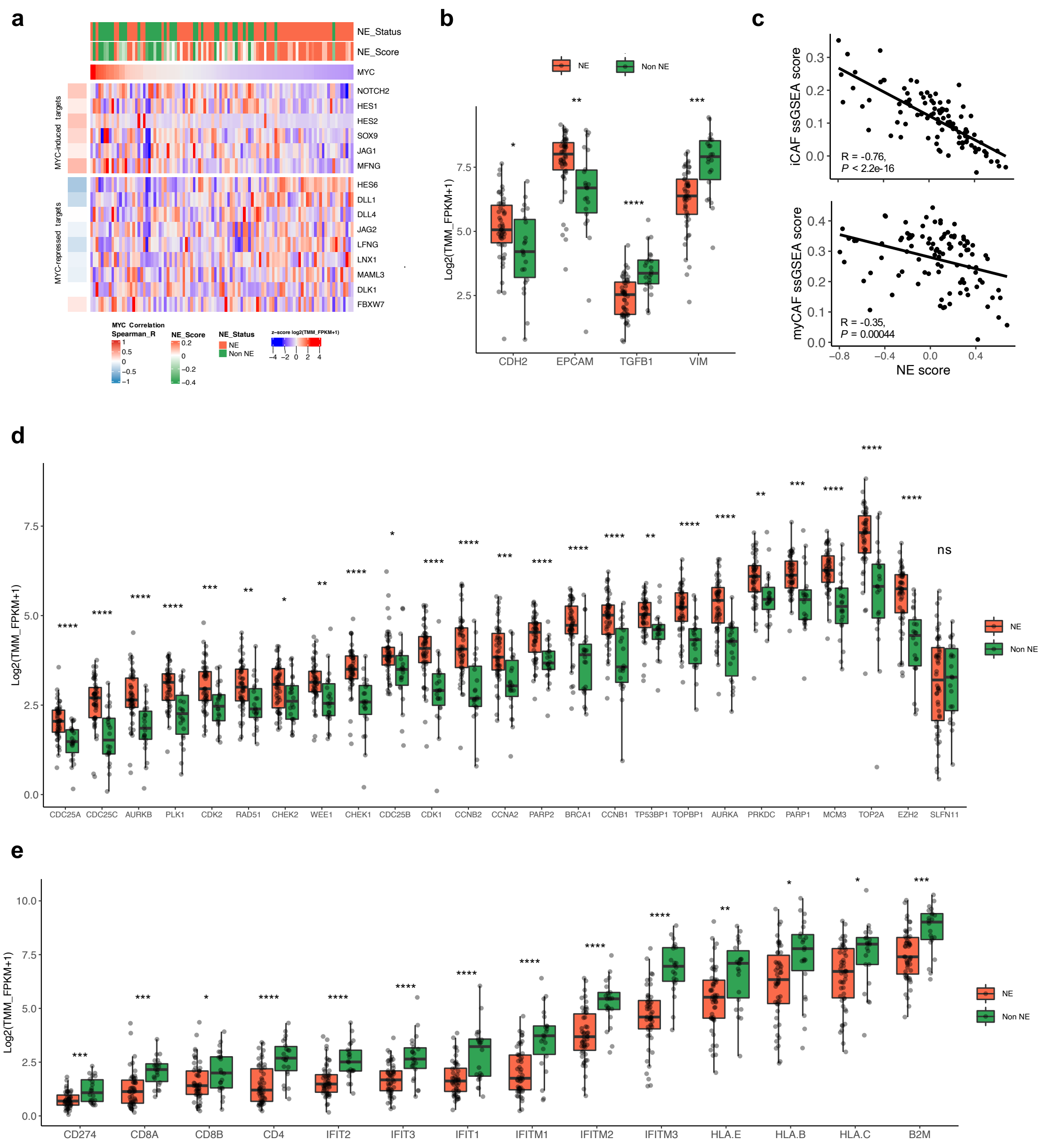

Supplementary Figure 11

(a) Heatmap visualizing Notch signaling pathway genes identified as MYC targets[31]. (b, d, e) Box plots show mRNA levels estimated in NE and non-NE tumors for selected (b) EMT, (d) DDR and (e) immune genes. Student's two-tailed unpaired *t* test with BH adjustment, *****P* < 0.0001, ****P* < 0.001, ***P* < 0.01, **P* < 0.05, ns, not significant (n=72 tumors). (c) Pearson correlation between the 50-gene signature score and inflammatory and myofibroblastic cancer-associated fibroblasts (iCAF and myCAF) signatures scores[50]. The Pearson's R values and *P*-values are indicated. All box plots indicate the inter-quartile range (IQR), the middle line corresponds to the median, and the upper and lower whiskers represent observations within 1.5*IQR (Q3 + 1.5*IQR or Q1 − 1.5*IQR). Abbreviations: NE: neuroendocrine differentiation; TMM: Trimmed Mean of M-values; FPKM: Fragments Per Kilobase of Exon Per Million Fragments Mapped.

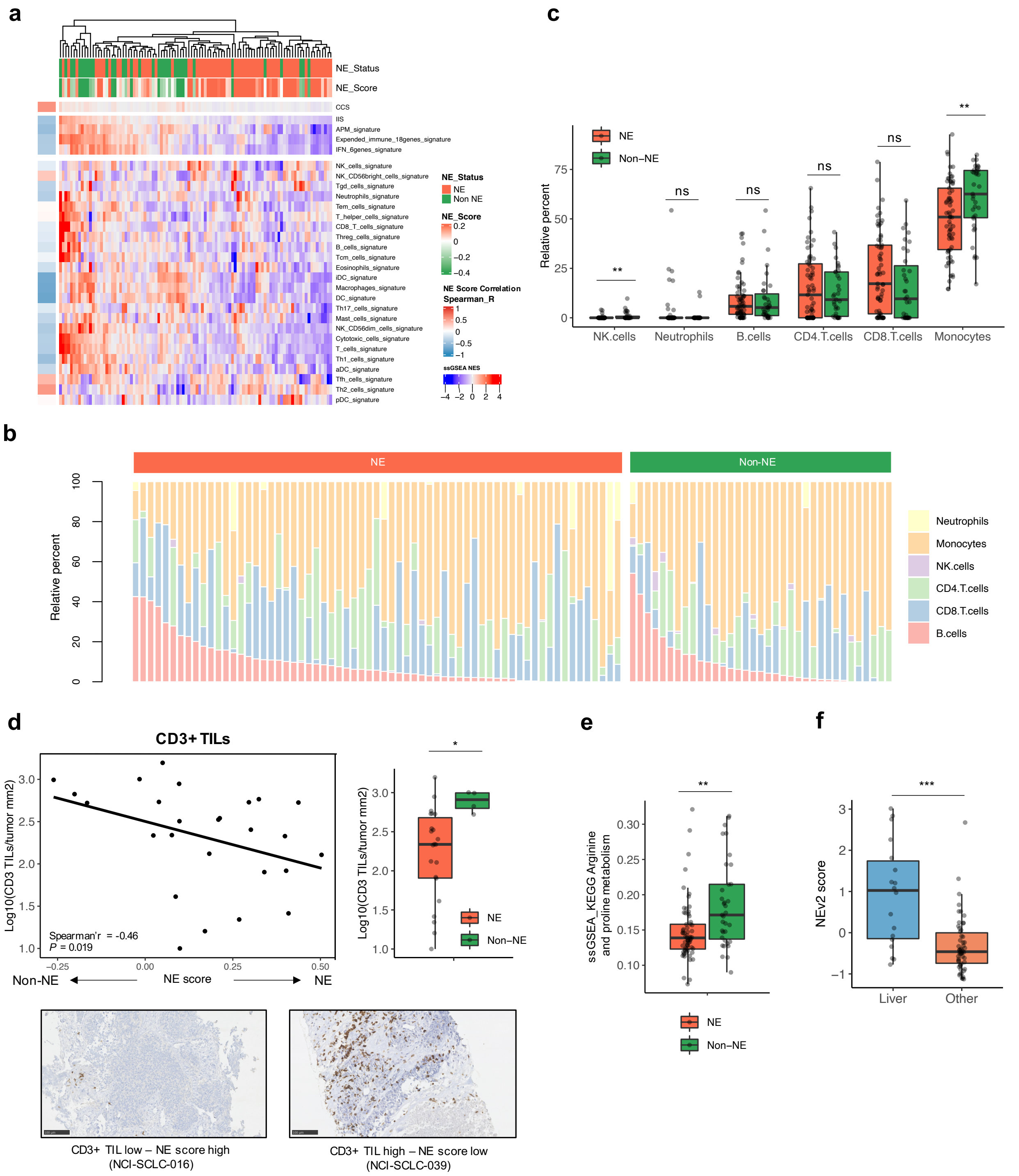
a
NE_Status
NE_Score
CCS
IIS
APM_signature
Expended_immune_18genes_signature
IFN_6genes_signature
NK_cells_signature
NK_CD56bright_cells_signature
Tgd_cells_signature
Neutrophils_signature
Tem_cells_signature
T_helper_cells_signature
CD8_T_cells_signature
Threg_cells_signature
B_cells_signature
Tcm_cells_signature
Eosinophils_signature
iDC_signature
Macrophages_signature
DC_signature
Th17_cells_signature
Mast_cells_signature
NK_CD56dim_cells_signature
Cytotoxic_cells_signature
T_cells_signature
Th1_cells_signature
aDC_signature
Tfh_cells_signature
Th2_cells_signature
pDC_signature
NE_Status
NE
Non NE
NE_Score
0.2
0
−0.2
−0.4
NE Score Correlation
Spearman_R
1
0.5
0
−0.5
−1
ssGSEA NES
−4 −2 0 2 4
c
NE
Non−NE
Relative percent
0
25
50
75
**
ns
ns
ns
ns
**
NK.cells
Neutrophils
B.cells
CD4.T.cells
CD8.T.cells
Monocytes
b
NE
Non-NE
Relative percent
0
20
40
60
80
100
Neutrophils
Monocytes
NK.cells
CD4.T.cells
CD8.T.cells
B.cells
d
CD3+ TILs
Log10(CD3 TILs/tumor mm2)
1.0
1.5
2.0
2.5
3.0
Spearman'r = -0.46
P = 0.019
−0.25
0.00
0.25
0.50
Non-NE
NE score
NE
*
NE
Non−NE
CD3+ TIL low – NE score high
(NCI-SCLC-016)
CD3+ TIL high – NE score low
(NCI-SCLC-039)
e
ssGSEA_KEGG Arginine
and proline metabolism
0.10
0.15
0.20
0.25
0.30
**
NE
Non−NE
f
NEv2 score
−1
0
1
2
3
***
Liver
Other

Supplementary Figure 12
(a) Heatmap visualizing cell cycle signature (CCS)[84], antigen processing machinery (APM)[63], immune infiltration score (IIS)[60,61], interferon-gamma (IFNγ) and expanded immune gene signatures scores[62]. (b) CIBERSORT analysis[25] of immune cell subsets[64] grouped by NE subtype. (c) Relative proportion of immune cells within NE and non-NE tumors based on CIBERSORT deconvolution. Two-tailed Mann-Whitney U-test, $**P < 0.01$ (ranging from P = 0.0024 to 0.0044), ns, not significant (n= 100 tumors). (d) Correlation between the 50-gene signature score and the intratumoral CD3+ TILs. The Spearman's R values and *P*-values are indicated. Box plot showing the distribution of the CD3+ TILs between NE and non-NE tumors is shown on the right. Two-tailed Mann-Whitney U-test, $*P = 0.013$ (n=26 tumors). Representative images of IHC staining for CD3+ TILs are shown for each category (bottom left = low, bottom right = high; observations were repeated independently 2 times). (e) Box plot showing the distribution of ssGSEA scores of the KEGG arginine and proline metabolism pathway between NE and non-NE tumors. Two-tailed Mann-Whitney U-test, $***P = 0.0011$ (n= 100 tumors). (f) Box plot showing the distribution of NEv2-like scores between liver and other biopsy sites. Two-tailed Mann-Whitney U-test, $***P = 0.00014$ (n= 72 patients). All box plots indicate the inter-quartile range (IQR), the middle line corresponds to the median, and the upper and lower whiskers represent observations within 1.5*IQR (Q3 + 1.5*IQR or Q1 − 1.5*IQR). Abbreviations: NE: neuroendocrine differentiation; TILs: tumor infiltrating lymphocytes; NES: normalized enrichment score.

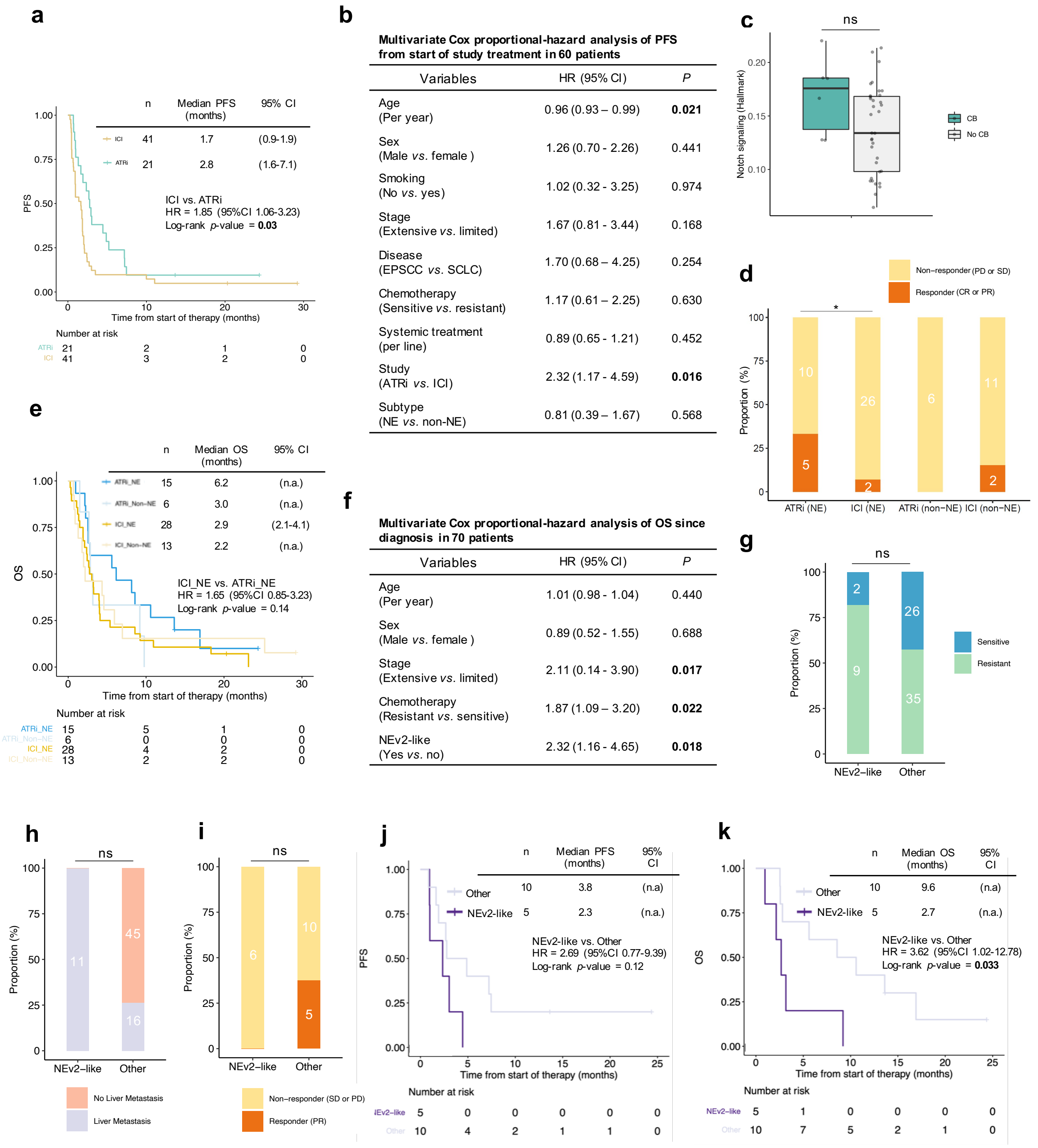

b Multivariate Cox proportional-hazard analysis of PFS from start of study treatment in 60 patients

Variables	HR (95% CI)	*P*
Age (Per year)	0.96 (0.93 – 0.99)	**0.021**
Sex (Male *vs.* female)	1.26 (0.70 - 2.26)	0.441
Smoking (No *vs.* yes)	1.02 (0.32 - 3.25)	0.974
Stage (Extensive *vs.* limited)	1.67 (0.81 - 3.44)	0.168
Disease (EPSCC *vs.* SCLC)	1.70 (0.68 – 4.25)	0.254
Chemotherapy (Sensitive *vs.* resistant)	1.17 (0.61 – 2.25)	0.630
Systemic treatment (per line)	0.89 (0.65 - 1.21)	0.452
Study (ATRi *vs.* ICI)	2.32 (1.17 - 4.59)	**0.016**
Subtype (NE *vs.* non-NE)	0.81 (0.39 – 1.67)	0.568

f Multivariate Cox proportional-hazard analysis of OS since diagnosis in 70 patients

Variables	HR (95% CI)	*P*
Age (Per year)	1.01 (0.98 - 1.04)	0.440
Sex (Male *vs.* female)	0.89 (0.52 - 1.55)	0.688
Stage (Extensive *vs.* limited)	2.11 (0.14 - 3.90)	**0.017**
Chemotherapy (Resistant *vs.* sensitive)	1.87 (1.09 – 3.20)	**0.022**
NEv2-like (Yes *vs.* no)	2.32 (1.16 - 4.65)	**0.018**

Supplementary Figure 13
(a) Kaplan-Meier curves and (b) multivariate Cox proportional-hazard regression analyses of PFS from start of study treatment in all comers. (c) Comparison of ssGSEA scores for Hallmark NOTCH signaling pathway in tumors of ICI-treated patients with or without CB. Two-tailed Mann-Whitney U-test, not significant (n=41 patients). (d) Overall response rate from ATR inhibition and immunotherapy categorized by NE subtypes. Two-tailed chi-square, *P = 0.027. (e) Kaplan-Meier curves of OS from start of treatment in patients treated with ATRi and ICI categorized by subtypes. HR and log-rank p-values are indicated (n=62 patients). (f) Multivariate Cox proportional-hazard regression analyses of OS since diagnosis in all comers. (g, h) Proportion of patients with NEv2-like tumors and (g) platinum resistant disease or (h) liver metastasis compared to patients with other tumor subtypes. Two-tailed chi-square, ns, not significant (n=72 patients). (i) Proportion of patients with NEv2-like tumors who achieved partial response with ATR inhibition compared to patients with other tumor subtypes. Two-tailed chi-square, not significant (n=72 patients). (j, k) Kaplan-Meier curves of (j) PFS and (k) OS from start of ATR treatment in patients with NEv2-like subtype vs. other. HR and log-rank p-values are indicated. Box plots indicate the inter-quartile range (IQR), the middle line corresponds to the median, and the upper and lower whiskers represent observations within 1.5*IQR (Q3 + 1.5*IQR or Q1 − 1.5*IQR). *P*-values marked with bold indicate statistical significance. Abbreviations: NE: neuroendocrine differentiation; SCLC: small cell lung cancer; OS: overall survival; PFS: progression free survival; HR: hazard ratio; CI: confidence interval; ATRi: ataxia telangiectasia and Rad3-related inhibitor; ICI: immune checkpoint inhibitor; CB: clinical benefit; PR: partial response; SD: stable disease; PD: progressive disease.

Supplementary Tables:

Supplementary Table 1: Patient characteristics
Supplementary Table 2: Patient tumor pathological characteristics
Supplementary Table 3: Patient clinical characteristics
Supplementary Table 4: Gene sets for the 50-, 10- and 70-gene signatures
Supplementary Table 5: Characteristics of patient tumors and their matched patient-derived xenografts (PDXs) counterparts
Supplementary Table 6: Upregulated pathways in tumors with NEv2-like subtype and in liver metastasis

Supplementary Table 1. Patient characteristics

All	Total (n=72)	NE (n=50, 69.4%)	Non-NE (n=22, 30.6%)	*P* value	ATRi (n=27, 37.5%)	ICI (n=42, 58.3%)	*P* value
Median age at diagnosis	62 (29-86)	61 (37-86)	65 (29-84)	0.81	61 (29-77)	62 (41-86)	0.057
Median age at biopsy	63 (29-86)	62 (41-86)	66 (29-84)	0.83	61 (29-79)	63 (41-86)	**0.043**
Median time from diagnosis to biopsy (months)	8 (0-47)	7 (0-47)	8 (0-18)	0.46	6 (0-47)	9 (0-42)	0.28
Biopsy timepoint							
Initial diagnosis	14 (19.4%)	11 (22%)	3 (13.6%)	0.53	8 (29.6%)	1 (2.4%)	**0.0017**
After recurrence	57 (79.2%)	38 (76%)	19 (86.4%)		19 (70.4%)	41 (97.6%)	
NA	1 (1.4%)	1 (2%)	-		-	-	
Sex							
Male	35 (48.6%)	25 (50%)	10 (45.5%)	0.8	13 (48.1%)	19 (45.2%)	1
Female	36 (50%)	25 (48%)	12 (54.5%)		14 (51.9%)	22 (52.4%)	
NA	1 (1.4%)	1 (2%)	-		-	1 (2.4%)	
Ancestry							
Caucasian	64 (88.9%)	46 (92%)	18 (81.8%)	0.36	25 (92.6%)	37 (88.1%)	1
Afrian American	6 (8.3%)	3 (6%)	3 (13.6%)		2 (7.4%)	3 (7.1%)	
Asian	1 (1.4%)	-	1 (4.5%)		-	1 (2.4%)	
NA	1 (1.4%)	1 (2%)	-		-	1 (2.4%)	
Histology							
SCLC	62 (86.1%)	44 (88%)	18 (81.8%)	0.85	20 (74.1%)	41 (97.6%)	1
†EPSCC	7 (9.7%)	4 (8%)	3 (13.6%)		6 (22.2%)	-	
EGFRmt-trSCLC	3 (4.2%)	2 (4%)	1 (4.5%)		1 (3.7%)	1 (2.4%)	
Stage at diagnosis							
Limited stage	18 (25%)	12 (24%)	6 (27.3%)	0.77	10 (37%)	7 (16.7%)	0.085
Extensive stage	54 (75%)	38 (76%)	16 (72.7%)		17 (63%)	35 (83.3%)	
Smoking history							
Never	8 (11.1%)	3 (6%)	5 (22.7%)	0.098	4 (14.8%)	2 (4.8%)	0.21
Current/former	62 (86.1%)	45 (90%)	17 (77.3%)		23 (85.2%)	38 (90.5%)	
NA	2 (34.7%)	2 (4%)	-		-	2 (4.8%)	
Platinum free duration (days)	62 (0-868)	72 (0-868)	26 (0-442)	0.45	68 (0-406)	66 (0-442)	0.64
Platinum sensitivity							
Sensitive	28 (38.9%)	17 (34%)	11 (50%)	0.29	7 (25.9%)	10 (23.8%)	1
Resistant	44 (61.1%)	33 (66%)	11 (50%)		20 (74.1%)	32 (76.2%)	
No. of systemic treatment	2 (1-6)	2 (1-4)	3 (1-6)	**0.02**	3 (2-5)	2 (1-6)	**3.40E-05**
Metastasis history							
Liver	26 (36%)	16 (32%)	10 (45.5%)	0.35	16 (59.3%)	9 (21.4%)	0.75
Brain	17 (23.6%)	14 (28%)	3 (13.6%)		10 (37%)	8 (19%)	
NA	25 (34.7%)	18 (36%)	7 (31.8%)		-	24 (57.1%)	
Cohort							
NCI	47 (65.3%)	32 (64%)	15 (68.2%)	0.79	27 (100%)	18 (41.9%)	
Rochester	25 (34.7%)	18 (36%)	7 (31.8%)		-	25 (58.1%)	
Trial							
ATRi	22 (30.6%)	15 (30%)	7 (31.8%)	0.93	22 (81.5%)	-	
ICI	37 (51.4%)	24 (48%)	13 (59.1%)		-	37 (88.1%)	
ATRi + ICI	5 (6.9%)	5 (10%)	.		5 (18.5%)	5 (11.9%)	
None	8 11.1%)	6 (12%)	2 (9.1%)		-	-	
NE Subtype							
NE	50 (69.4%)	-	-		20 (74.1%)	29 (69%)	0.79
Non-NE	22 (30.6%)	-	-		7 (25.9%)	13 (31%)	

Description:

†EPSCC: prostate, rectum, ovary, bladder, cervix and subglottis

P-values from Fisher's test and t-test. P-values marked with bold indicate statistical significance.

Supplementary Table 2. Patient tumor pathological characteristics

Sample id	Patient id	Biopsy site	Biopsy date	Biopsy type	Synaptophysin	Chromogranin
NCI-EP-096	P-065	Right adrenal gland	27/07/2016	Excision	+	+
NCI-SCLC-001	P-001	Liver	20/10/2016	Core biopsy	+	+
NCI-SCLC-002	P-001	Liver	20/10/2016	Core biopsy	+	+
NCI-SCLC-003	P-002	Liver	05/04/2016	Core biopsy	+	+
NCI-SCLC-004	P-003	Liver	25/05/2016	Core biopsy	NA	NA
NCI-SCLC-005	P-003	Liver	25/05/2016	Core biopsy	NA	NA
NCI-SCLC-006	P-004	Liver	13/01/2017	Core biopsy	+	-
NCI-SCLC-007	P-005	Left adrenal mass	21/03/2017	Core biopsy	+	+
NCI-SCLC-008	P-006	Left lung	04/08/2016	Core biopsy	+	+
NCI-SCLC-009	P-007	Left upper lobe	08/06/2016	Excision	+	-
NCI-SCLC-010	P-008	Right chest wall	20/09/2017	Core biopsy	+	NA
NCI-SCLC-011	P-008	Right chest wall	20/09/2017	Core biopsy	+	NA
NCI-SCLC-012	P-008	Right pleural soft tissue	17/10/2017	Core biopsy	+	+
NCI-SCLC-013	P-009	Mediastinal mass	31/10/2017	Core biopsy	+	-
NCI-SCLC-014	P-009	Mediastinal mass	15/11/2017	Core biopsy	NA	NA
NCI-SCLC-015	P-010	Liver	27/10/2017	Core biopsy	+	-
NCI-SCLC-016	P-010	Liver	13/11/2017	Core biopsy	NA	NA
NCI-SCLC-017	P-011	Left supraclavicular LN	31/10/2017	Core biopsy	+	-
NCI-SCLC-018	P-011	Left supraclavicular LN	14/11/2017	Core biopsy	NA	NA
NCI-SCLC-019	P-012	Left adrenal	16/12/2016	Core biopsy	NA	NA
NCI-SCLC-020	P-013	Liver	02/02/2018	Core biopsy	NA	NA
NCI-SCLC-021	P-013	Liver	16/02/2018	Core biopsy	NA	NA
NCI-SCLC-022	P-014	Subcarinal mass	13/02/2017	Core biopsy	+	+
NCI-SCLC-023	P-015	Liver	12/02/2018	Core biopsy	+	+
NCI-SCLC-024	P-016	Right middle lobe lung	27/07/2017	Excision	+	+
NCI-EP-089	P-066	Prostate	21/04/2016	Excision	+	+
NCI-EP-090	P-067	Right inguinal LN	05/12/2017	Core biopsy	+	+
NCI-SCLC-025	P-017	Mediastinal mass	20/03/2016	Core biopsy	+	+
NCI-EP-091	P-068	Right Pelvic mass	10/04/2018	Core biopsy	NA	NA
NCI-SCLC-026	P-018	Liver	02/02/2018	Core biopsy	+	NA
NCI-EP-092	P-069	Bladder	16/11/2015	Core biopsy	+	+
NCI-SCLC-027	P-019	Liver	30/03/2018	Core biopsy	NA	NA
NCI-EP-093	P-070	Uterine cervix	17/08/2016	Core biopsy	+	-
NCI-SCLC-028	P-020	Left neck LN	04/04/2018	Excision	+	NA
NCI-SCLC-029	P-020	Left neck LN	09/05/2018	Core biopsy	NA	NA
NCI-SCLC-030	P-021	Left lung/mediastinal	10/04/2018	Core biopsy	NA	NA
NCI-SCLC-031	P-022	Left inguinal LN	01/06/2018	Core biopsy	+	NA
NCI-EP-097	P-071	Right upper lung lobe	30/05/2018	Core biopsy	+	NA
NCI-SCLC-032	P-023	Right supraclavicular LN	12/09/2018	Core biopsy	NA	NA
NCI-SCLC-033	P-024	Liver	14/06/2018	Core biopsy	NA	NA
NCI-SCLC-034	P-025	Liver	22/02/2019	Core biopsy	NA	NA
NCI-SCLC-035	P-026	Right adrenal gland	15/01/2019	Excision	NA	NA
NCI-SCLC-036	P-061	Left hilar/subcarinal/right paratracheal/right hilas LN	11/01/2019	FNAC	+	+

NCI-SCLC-037	P-061	Left hilar/subcarinal/right paratracheal/right hilas LN	11/01/2019	FNAC	+	+
NCI-SCLC-038	P-062	Left upper lobe lung	22/03/2019	FNAC	+	+
NCI-EP-098	P-072	Liver	04/05/2018	Excision	+	NA
NCI-EP-099	P-072	Liver	04/05/2018	Excision	+	NA
NCI-SCLC-039	P-027	Right neck LN	15/12/2016	Core biopsy	NA	NA
NCI-SCLC-040	P-027	Right neck LN	15/12/2016	Core biopsy	NA	NA
NCI-SCLC-041	P-027	Left cerebellar lesion	20/11/2017	Excision	+	-
NCI-SCLC-042	P-053	Left upper lobe mass	25/10/2011	Excision	+	+
NCI-SCLC-043	P-013	Liver	22/09/2017	Core biopsy	+	NA
NCI-EP-094	P-070	Liver	24/05/2018	Core biopsy	NA	NA
NCI-SCLC-044	P-024	Liver	11/06/2018	Core biopsy	+	+
NCI-EP-095	P-064	Pelvic mass	07/10/2019	Core biopsy	+	+
NCI-SCLC-045	P-054	Liver	06/03/2019	Core biopsy	NA	NA
NCI-SCLC-046	P-058	Liver	18/03/2019	Core biopsy	NA	NA
NCI-SCLC-047	P-059	Liver	11/04/2019	Core biopsy	+	+
NCI-SCLC-048	P-054	Liver	18/01/2019	Core biopsy	NA	NA
NCI-SCLC-049	P-057	Liver	11/02/2019	Core biopsy	NA	NA
NCI-SCLC-050	P-057	Liver	11/02/2019	Core biopsy	NA	NA
NCI-EP-100	P-063	Subglottis	23/03/2018	FNAC	+	+
NCI-SCLC-051	P-060	Lung	07/09/2018	Core biopsy	+	-
NCI-SCLC-052	P-058	Liver	28/11/2018	Core biopsy	-	-
NCI-SCLC-053	P-055	Right supraclavicular LN	13/10/2017	Core biopsy	NA	NA
NCI-SCLC-054	P-056	Right frontal brain lesion	30/11/2017	Excision	+	-

Description:

**Sample id**	Identification number for each sample
**Patient id**	Identification number for each patient
**Biopsy site**	Biopsy site of corresponding clinical tumors
**Biopsy date**	Biopsy date of corresponding clinical tumors
**Biopsy type**	Biopsy type of corresponding clinical tumors
**Chromogranin**	Intensity of chromogranin immunohistochemistry staining
**Synaptophysin**	Intensity of synaptophysin immunohistochemistry staining

Abbreviations: NA: not available; FNAC: fine needle aspiration core biopsy; LN: lymph node

Supplementary Table 3. Patient clinical characteristics

Sample id	Patient id	Trial_ ATRi	Trial_IC+ PARPi	Trial_IC	Disease	Age at diagnosis	SCLC staging/initial diagnosis	Smoking history	Platinum sensitivity	Number of systemic therapy	IO prior to biopsy	Mortality status	OS since diagnosis	Age at biopsy timepoint	Duration from diagnosis to biopsy	Biopsy timepoint
NCI-SCLC-001	P-001	No	Yes	No	SCLC	69	Extensive stage	Yes	resistant	3	No	Dead	8.76923077	70	7.483516484	Relapse
NCI-SCLC-002	P-001	No	Yes	No	SCLC	69	Extensive stage	Yes	resistant	3	No	Dead	8.76923077	70	7.483516484	Relapse
NCI-SCLC-003	P-002	No	Yes	No	SCLC	71	Extensive stage	Yes	resistant	3	No	Dead	11.967033	72	7.78021978	Relapse
NCI-SCLC-004	P-003	No	Yes	No	SCLC	59	Extensive stage	No	resistant	2	No	Dead	10.6153846	60	9.230769231	Relapse
NCI-SCLC-005	P-003	No	Yes	No	SCLC	59	Extensive stage	No	resistant	2	No	Dead	10.6153846	60	9.230769231	Relapse
NCI-SCLC-006	P-004	No	Yes	No	SCLC	72	Extensive stage	Yes	resistant	2	No	Dead	7.41758242	72	6.758241758	Relapse
NCI-SCLC-007	P-005	No	Yes	No	SCLC	57	Limited stage	Yes	resistant	2	No	Dead	11.5714286	57	7.285714286	Relapse
NCI-SCLC-008	P-006	No	Yes	No	SCLC	41	Extensive stage	No	sensitive	2	No	Dead	14.8351648	41	7.846153846	Relapse
NCI-SCLC-009	P-007	Yes	Yes	No	SCLC	57	Extensive stage	Yes	sensitive	3	Yes	Dead	18.1648352	59	18.16483516	Relapse
NCI-SCLC-010	P-008	No	Yes	No	SCLC	75	Extensive stage	Yes	resistant	2	No	Dead	8.07692308	75	5.208791209	Relapse
NCI-SCLC-011	P-008	No	Yes	No	SCLC	75	Extensive stage	Yes	resistant	2	No	Dead	8.07692308	75	5.208791209	Relapse
NCI-SCLC-012	P-008	No	Yes	No	SCLC	75	Extensive stage	Yes	resistant	2	Yes	Dead	8.07692308	75	6.098901099	Relapse
NCI-SCLC-013	P-009	Yes	Yes	No	SCLC	62	Limited stage	Yes	sensitive	3	No	Dead	20.5054945	63	17.20879121	Relapse
NCI-SCLC-014	P-009	Yes	Yes	No	SCLC	62	Limited stage	Yes	sensitive	3	Yes	Dead	20.5054945	63	17.7032967	Relapse
NCI-SCLC-015	P-010	No	Yes	No	SCLC	64	Limited stage	Yes	sensitive	3	No	Dead	23.0769231	65	18.49450549	Relapse
NCI-SCLC-016	P-010	No	Yes	No	SCLC	64	Limited stage	Yes	sensitive	3	Yes	Dead	23.0769231	65	19.05494505	Relapse
NCI-SCLC-017	P-011	No	Yes	No	SCLC	60	Extensive stage	Yes	sensitive	2	No	Dead	46.0879121	63	36.85714286	Relapse
NCI-SCLC-018	P-011	No	Yes	No	SCLC	60	Extensive stage	Yes	sensitive	2	Yes	Dead	46.0879121	63	37.31868132	Relapse
NCI-SCLC-019	P-012	No	Yes	No	SCLC	63	Extensive stage	Yes	resistant	3	No	Dead	15.1978022	63	0	Initial diagnosis
NCI-SCLC-020	P-013	Yes	Yes	No	SCLC	63	Extensive stage	Yes	resistant	3	No	Dead	13.0549451	64	10.61538462	Relapse
NCI-SCLC-021	P-013	Yes	Yes	No	SCLC	63	Extensive stage	Yes	resistant	3	Yes	Dead	13.0549451	64	11.07692308	Relapse
NCI-SCLC-043	P-013	Yes	Yes	No	SCLC	63	Extensive stage	Yes	resistant	3	No	Dead	13.0549451	64	6.230769231	Relapse
NCI-SCLC-022	P-014	Yes	No	No	SCLC	69	Limited stage	Yes	resistant	2	No	Dead	17.3406593	69	0	Initial diagnosis
NCI-SCLC-023	P-015	Yes	No	No	SCLC	57	Extensive stage	Yes	resistant	2	No	Dead	5.17582418	57	3.56043956	Relapse
NCI-SCLC-024	P-016	Yes	No	No	SCLC	64	Extensive stage	Yes	resistant	2	No	Dead	17.1758242	65	17.17582418	Relapse
NCI-SCLC-025	P-017	Yes	No	No	SCLC	53	Limited stage	Yes	resistant	3	No	Alive	51.2637363	53	0	Initial diagnosis
NCI-SCLC-026	P-018	No	No	No	SCLC	65	Extensive stage	Yes	resistant	1	No	Dead	6.42857143	65	0	Initial diagnosis
NCI-SCLC-027	P-019	Yes	No	No	SCLC	59	Extensive stage	Yes	resistant	2	No	Dead	6.13186813	59	3.32967033	Relapse
NCI-SCLC-028	P-020	No	Yes	No	SCLC	75	Limited stage	Yes	resistant	3	Yes	Dead	12.4285714	76	7.912087912	Relapse
NCI-SCLC-029	P-020	No	Yes	No	SCLC	75	Limited stage	Yes	resistant	3	Yes	Dead	12.4285714	76	9.065934066	Relapse
NCI-SCLC-030	P-021	Yes	Yes	No	SCLC	55	Extensive stage	Yes	resistant	4	No	Dead	24.2637363	56	5.934065934	Relapse
NCI-SCLC-031	P-022	Yes	Yes	No	SCLC	50	Limited stage	Yes	resistant	4	No	Dead	20.2417582	51	16.21978022	Relapse
NCI-SCLC-032	P-023	Yes	No	No	SCLC	58	Extensive stage	Yes	resistant	3	No	Dead	10.021978	59	7.417582418	Relapse
NCI-SCLC-033	P-024	Yes	No	No	SCLC	71	Extensive stage	Yes	resistant	2	Yes	Dead	6.75824176	71	5.868131868	Relapse
NCI-SCLC-044	P-024	Yes	No	No	SCLC	71	Extensive stage	Yes	resistant	2	Yes	Dead	6.75824176	71	5.769230769	Relapse
NCI-SCLC-034	P-025	Yes	No	No	SCLC	63	Extensive stage	No	resistant	3	Yes	Dead	13.6813187	63	4.384615385	Relapse
NCI-SCLC-035	P-026	Yes	No	No	SCLC	71	Extensive stage	Yes	sensitive	5	Yes	Dead	20.8681319	73	17.30769231	Relapse
NCI-SCLC-039	P-027	No	Yes	No	SCLC	54	Limited stage	Yes	resistant	6	No	Dead	39.2307692	55	14.04395604	Relapse
NCI-SCLC-040	P-027	No	Yes	No	SCLC	54	Limited stage	Yes	resistant	6	No	Dead	39.2307692	55	14.04395604	Relapse
NCI-SCLC-041	P-027	No	Yes	No	SCLC	54	Limited stage	Yes	resistant	6	Yes	Dead	39.2307692	56	25.25274725	Relapse
NCI-SCLC-063	P-028	No	No	Yes	SCLC	59	Extensive stage	Yes	sensitive	1	No	Dead	12.956044	60	11.86813187	Relapse
NCI-SCLC-064	P-029	No	No	Yes	SCLC	54	Extensive stage	Yes	resistant	2	No	Dead	23.5714286	56	18.1978022	Relapse
NCI-SCLC-065	P-029	No	No	Yes	SCLC	54	Extensive stage	Yes	resistant	2	No	Dead	23.5714286	56	18.1978022	Relapse
NCI-SCLC-066	P-030	No	No	Yes	SCLC	68	Extensive stage	Yes	resistant	2	No	Dead	17.0769231	69	15.16483516	Relapse
NCI-SCLC-067	P-031	No	No	Yes	SCLC	68	Extensive stage	Yes	sensitive	1	No	Dead	15.032967	69	9.098901099	Relapse
NCI-SCLC-068	P-032	No	No	Yes	SCLC	68	Extensive stage	Yes	sensitive	1	No	Dead	10.5494505	69	8.43956044	Relapse
NCI-SCLC-069	P-032	No	No	Yes	SCLC	68	Extensive stage	Yes	sensitive	1	No	Dead	10.5494505	69	8.43956044	Relapse

NCI-SCLC-070	P-032	No	No	Yes	SCLC	68	Extensive stage	Yes	sensitive	1	No	Dead	10.5494505	69	8.43956044	Relapse
NCI-SCLC-071	P-033	No	No	Yes	SCLC	71	Extensive stage	Yes	resistant	1	No	Dead	6.98901099	72	6.725274725	Relapse
NCI-SCLC-072	P-033	No	No	Yes	SCLC	71	Extensive stage	Yes	resistant	1	No	Dead	6.98901099	72	6.725274725	Relapse
NCI-SCLC-073	P-034	No	No	Yes	SCLC	84	Extensive stage	Yes	sensitive	1	No	Alive	38.3736264	84	9.164835165	Relapse
NCI-SCLC-074	P-035	No	No	Yes	SCLC	59	Extensive stage	Yes	resistant	1	No	Dead	6.62637363	60	6.263736264	Relapse
NCI-SCLC-075	P-035	No	No	Yes	SCLC	59	Extensive stage	Yes	resistant	1	No	Dead	6.62637363	60	6.263736264	Relapse
NCI-SCLC-076	P-036	No	No	Yes	SCLC	59	Extensive stage	Yes	sensitive	1	No	Dead	50.9010989	62	42.32967033	Relapse
NCI-SCLC-055	P-037	No	No	Yes	SCLC	61	Extensive stage	Yes	resistant	1	No	Dead	4.38461538	61	1.648351648	Relapse
NCI-SCLC-077	P-038	No	No	Yes	SCLC	56	Extensive stage	Yes	sensitive	2	No	Dead	12.0989011	57	10.87912088	Relapse
NCI-SCLC-078	P-039	No	No	Yes	SCLC	57	Extensive stage	NA	sensitive	2	No	Dead	51.4945055	59	28.35164835	Relapse
NCI-SCLC-079	P-039	No	No	Yes	SCLC	57	Extensive stage	NA	sensitive	2	No	Dead	51.4945055	59	28.35164835	Relapse
NCI-SCLC-080	P-040	No	No	Yes	SCLC	67	Extensive stage	Yes	sensitive	1	No	Dead	13.6153846	67	10.58241758	Relapse
NCI-SCLC-081	P-041	No	No	Yes	SCLC	58	Extensive stage	Yes	sensitive	1	No	Dead	18.1648352	59	7.21978022	Relapse
NCI-SCLC-082	P-041	No	No	Yes	SCLC	58	Extensive stage	Yes	sensitive	1	No	Dead	18.1648352	59	7.21978022	Relapse
NCI-SCLC-083	P-042	No	No	Yes	SCLC	62	Extensive stage	Yes	sensitive	1	No	Dead	8.50549451	63	7.747252747	Relapse
NCI-SCLC-056	P-043	No	No	Yes	SCLC	68	Extensive stage	Yes	sensitive	1	No	Dead	11.4725275	69	9.296703297	Relapse
NCI-SCLC-084	P-044	No	No	Yes	SCLC	75	Extensive stage	Yes	resistant	1	No	Dead	7.45054945	NA	NA	NA
NCI-SCLC-085	P-045	No	No	Yes	SCLC	56	Extensive stage	Yes	sensitive	2	No	Dead	12.0989011	57	10.12087912	Relapse
NCI-SCLC-086	P-045	No	No	Yes	SCLC	56	Extensive stage	Yes	sensitive	2	No	Dead	12.0989011	57	10.12087912	Relapse
NCI-SCLC-087	P-046	No	No	Yes	SCLC	59	Extensive stage	Yes	resistant	1	No	Dead	3.1978022	59	2.769230769	Relapse
NCI-SCLC-088	P-047	No	No	Yes	SCLC	71	Extensive stage	NA	sensitive	1	No	Dead	13.6813187	72	11.30769231	Relapse
NCI-SCLC-057	P-048	No	No	Yes	SCLC	54	Limited stage	Yes	resistant	2	No	Dead	20.967033	56	19.48351648	Relapse
NCI-SCLC-058	P-048	No	No	Yes	SCLC	54	Limited stage	Yes	resistant	2	No	Dead	20.967033	56	19.48351648	Relapse
NCI-SCLC-059	P-049	No	No	Yes	SCLC	60	Extensive stage	Yes	sensitive	2	No	Dead	14.2747253	60	12.49450549	Relapse
NCI-SCLC-060	P-050	No	No	Yes	SCLC	66	Extensive stage	Yes	sensitive	3	No	Dead	16.2197802	68	15.32967033	Relapse
NCI-SCLC-061	P-051	No	No	Yes	SCLC	86	Extensive stage	Yes	resistant	1	No	Dead	5.83516484	86	3.923076923	Relapse
NCI-SCLC-062	P-052	No	No	Yes	SCLC	61	Extensive stage	Yes	resistant	1	No	Dead	9.62637363	62	5.637362637	Relapse
NCI-SCLC-042	P-053	No	No	No	SCLC	52	Limited stage	Yes	sensitive	2	No	Dead	45.2967033	55	31.08791209	Relapse
NCI-SCLC-045	P-054	Yes	No	No	SCLC	65	Limited stage	Yes	resistant	4	Yes	Dead	20.5054945	66	14.50549451	Relapse
NCI-SCLC-048	P-054	Yes	No	No	SCLC	65	Limited stage	Yes	resistant	4	No	Dead	20.5054945	66	12.95604396	Relapse
NCI-SCLC-053	P-055	Yes	No	No	SCLC	65	Limited stage	Yes	sensitive	2	No	Dead	21.6263736	65	0	Initial diagnosis
NCI-SCLC-054	P-056	Yes	No	No	SCLC	66	Extensive stage	Yes	resistant	3	No	Alive	30.989011	66	0	Initial diagnosis
NCI-SCLC-049	P-057	Yes	No	No	SCLC	61	Extensive stage	Yes	resistant	2	No	Dead	9.75824176	61	7.582417582	Relapse
NCI-SCLC-050	P-057	Yes	No	No	SCLC	61	Extensive stage	Yes	resistant	2	No	Dead	9.75824176	61	7.582417582	Relapse
NCI-SCLC-046	P-058	Yes	No	No	SCLC	75	Extensive stage	Yes	resistant	2	No	Dead	6.85714286	75	3.626373626	Relapse
NCI-SCLC-052	P-058	Yes	No	No	SCLC	75	Extensive stage	Yes	resistant	2	No	Dead	6.85714286	75	0	Initial diagnosis
NCI-SCLC-047	P-059	Yes	No	No	SCLC	50	Extensive stage	Yes	sensitive	4	Yes	Dead	20.3736264	51	14.8021978	Relapse
NCI-SCLC-051	P-060	No	No	No	SCLC	70	Extensive stage	Yes	resistant	4	No	Dead	14.4065934	70	0	Initial diagnosis
NCI-SCLC-036	P-061	No	No	No	SCLC	62	Limited stage	Yes	sensitive	2	No	Alive	16.3846154	62	0	Initial diagnosis
NCI-SCLC-037	P-061	No	No	No	SCLC	62	Limited stage	Yes	sensitive	2	No	Alive	16.3846154	62	0	Initial diagnosis
NCI-SCLC-038	P-062	No	No	No	SCLC	66	Extensive stage	Yes	resistant	1	No	Alive	6.23076923	66	0	Initial diagnosis
NCI-EP-100	P-063	No	No	No	EP_subgl	63	Limited stage	No	sensitive	2	No	Dead	20.6373626	64	4.714285714	Relapse
NCI-EP-095	P-064	Yes	No	No	EP_cervi	37	Limited stage	No	sensitive	4	No	Dead	53.2417582	41	46.81318681	Relapse
NCI-EP-096	P-065	No	No	No	EP_EGFR	60	Extensive stage	No	resistant	3	No	Dead	12.8241758	60	0	Initial diagnosis
NCI-EP-089	P-066	Yes	No	No	EP_prost	74	Limited stage	Yes	resistant	4	No	Dead	13.6813187	74	0	Initial diagnosis
NCI-EP-090	P-067	Yes	No	No	EP_rectur	77	Extensive stage	Yes	resistant	3	Yes	Dead	29.7032967	79	21.23076923	Relapse
NCI-EP-091	P-068	Yes	No	No	EP_ovary	37	Limited stage	No	resistant	4	No	Dead	7.31868132	37	5.802197802	Relapse
NCI-EP-092	P-069	Yes	No	No	EP_bladd	52	Limited stage	Yes	sensitive	4	No	Dead	39.032967	52	0	Initial diagnosis
NCI-EP-093	P-070	Yes	No	No	EP_cervi	29	Extensive stage	Yes	resistant	5	No	Dead	27.8241758	29	0	Initial diagnosis
NCI-EP-094	P-070	Yes	No	No	EP_cervi	29	Extensive stage	Yes	resistant	5	Yes	Dead	27.8241758	31	21.26373626	Relapse
NCI-EP-097	P-071	No	Yes	No	EP_EGFR	69	Extensive stage	Yes	resistant	2	No	Alive	27.8571429	69	2.835164835	Relapse
NCI-EP-098	P-072	Yes	No	No	EP_EGFR	39	Extensive stage	No	resistant	3	No	Dead	4.58241758	39	1.186813187	Relapse
NCI-EP-099	P-072	Yes	No	No	EP_EGFR	39	Extensive stage	No	resistant	3	No	Dead	4.58241758	39	1.186813187	Relapse

Description:

Trial_ATRi: Whether patient was enrolled in a trial of topotecan + ATR inhibitor berzosertib (NCT02487095)

Trial_IO_PARPi: Whether patient was enrolled in a trial of PARP inhibitor olaparib + immune checkpoint inhibitor durvalumab (NCT02484404)

Trial_IO: Whether patient was enrolled in a trial of immune checkpoint inhibitor nivolumab (Rochester cohort)

Disease: SCLC: Small cell lung cancer; EP: extrapulmonary small cell cancer. Organs after EP indicates primary site for extrapulmonary small cell cancer

Age at diagnosis: Age at diagnosis in years

SCLC staging/initial diagnosis: Clinical stage at diagnosis based on Veteran Administration Lung Group

Smoking history: No: patients having smoked < 100 cigarettes in their lifetime; Yes: patients having smoked ≥ 100 cigarettes in their lifetime

Platinum sensitivity: Sensitive: disease progression or recurrence in ≥ 90 days after platinum–based chemotherapy; resistant: disease progression or recurrence in < 90 days after platinum–based chemotherapy

Number of systemic therapy: Number of systemic therapy prior to study entry

Immunotherapy prior to biopsy: Whether patient had received immunotherapy prior to biopsy

Surgery for SCLC: Whether patient had surgical intervention for SCLC/EP

Liver metastasis: Whether patient had liver metastasis, either at diagnosis or thereafter

Brain metastasis: Whether patient had brain metastasis, either at diagnosis or thereafter

Mortality status: Alive: the patient was alive at the date of cutoff; Dead: the patient was dead at the date of cutoff

Overall survival since diagnosis: Time in months between diagnosis of SCLC and either date of patient death or date of cutoff

Age at biopsy timepoint: Age at biopsy timepoint in years

Duration from diagnosis to biopsy: Time in months between diagnosis of SCLC and biopsy

Biopsy timepoint: Initial diagnosis: biopsy was done at SCLC diagnosis; Relapse: biopsy was done after recurrence/relapse with platinum-based chemotherapy or thereafter

Abbreviations:

NA: not available; SCLC: small cell lung cancer; EP: extrapulmonary small cell cancer; IO: immune checkpoint; ATR: ataxia telangiectasia and Rad3-related; PARP: poly (ADP ribose) polymerase; OS: overall survival

Supplementary Table 4. Gene sets for the 50-, 10- and 70-gene signatures

Gene signature	Gene up-regulated in NE	Gene down-regulated in NE	Reference
10-genes	SCG3, CHGA, CHGB, CHRNB2, PCSK1, ELAVL4, ENO2, SCN3A, SYP, NKX2-1	NA	Bluemn et al.[16]
50-genes	BEX1, ASCL1, INSM1, CHGA, TAGLN3, KIF5C, CRMP1, SCG3, SYT4, RTN1, MYT1, SYP, KIF1A, TMSB15A, SYN1, SYT11, RUNDC3A, TFF3, CHGB, FAM57B, SH3GL2, BSN, SEZ6, TMSB15B, CELF3	RAB27B, TGFBR2, SLC16A5, S100A10, ITGB4, YAP1, LGALS3, EPHA2, S100A16, PLAU, ABCC3, ARHGDIB, CYR61, PTGES, CCND1, IFITM2, IFITM3, AHNAK, CAV2, TACSTD2, TGFBI, EMP1, CAV1, ANXA1, MYOF	Zhang et al.[15]
70-genes	ASXL3, CAND2, ETV5, GPX2, JAKMIP2, KIAA0408, SOGA3, TRIM9, BRINP1, C7orf76, GNAO1, KCNB2, KCND2, LRRC16B, MAP10, NRSN1, PCSK1, PROX1, RGS7, SCG3, SEC11C, SEZ6, ST8SIA3, SVOP, SYT11, AURKA, DNMT1, EZH2, MYCN	RHGAP8, CATSPERB, EFNA4, EPN3, EVPL, HOXB13, KLK3, KLK4, LMAN1L, NKX3-1, OPHN1, PIEZO1, PRR5-ARHGAP8, PSCA, RAB27B, RGS10, RIPK2, SLC25A37, SLC44A4, TC2N, UPK2, RB1, AR, CCND1, CIITA, CREBBP, CSDE1, CYLD, DICER1, FHIT, FOXP1, HERPUD1, MMP2, MYH9, NUP93, PAX8, RBBP6, TRIM33, GATA2, MAPKAPK3,	Beltran et al.[17]

Supplementary Table 5. Characteristics of patient tumors and their matched patient-derived xenograft (PDX) counterparts

Sample id	Patient id	Age at diagnosis	SCLC staging/initial diagnosis	Smoking history	Platinum sensitivity	IC prior to biopsy	Biopsy site	Biopsy type	Duration from diagnosis to biopsy	Chromogranin _PDX	Synaptophysin _PDX
NCI-SCLC-045	P-054	65	Limited stage	Yes	resistant	Yes	Liver	Core biopsy	14.50549451	+	+
NCI-SCLC-046	P-058	75	Extensive stage	Yes	resistant	No	Liver	Core biopsy	3.626373626	-	+
NCI-PDX-001	P-073	69	Limited stage	Yes	sensitive	Yes	Liver	Core biopsy	11.17582418	+	+
NCI-PDX-002	P-074	63	Extensive stage	Yes	resistant	Yes	Liver	Core biopsy	7.714285714	+	+++
NCI-PDX-003	P-075	64	Limited stage	Yes	resistant	No	Retroperitoneal LN	Core biopsy	8.604395604	+	+++
NCI-PDX-004	P-076	47	Limited stage	No	resistant	Yes	Liver	Core biopsy	4.648351648	+++	+++

Description:

Sample id: Identification number for each sample

Patient id: Identification number for each patient

Age at diagnosis: Age at diagnosis in years

SCLC staging/initial diagnosis: Clinical stage at diagnosis based on Veteran Administration Lung Group

Smoking history: No: patients having smoked < 100 cigarettes in their lifetime; Yes: patients having smoked ≥ 100 cigarettes in their lifetime

Platinum sensitivity: Sensitive: PD or recurrence > 90 days after platinum–based CT initiation; resistant: disease progression or recurrence < 90 days after platinum–based CT initiation

Immunotherapy prior to biopsy: Whether patient had received immunotherapy prior to biopsy

Biopsy site: Biopsy site of corresponding clinical tumors

Biopsy type: Biopsy type of corresponding clinical tumors

Duration from diagnosis to biopsy: Time in months between diagnosis of SCLC and biopsy

Chromogranin_PDX: Intensity of chromogranin immunohistochemistry staining in PDX

Synaptophysin_PDX: Intensity of synaptophysin immunohistochemistry staining in PDX

Abbreviations: PDX: Patient-delivered xenograft**;** SCLC: small cell lung cancer; IC: immune checkpoint; LN: lymph node; PD: progressive disease; CT: chimiotherapy; NA: not available

Supplementary Table 6. Upregulated pathways in tumors with NEv2-like subtype and in liver metastasis

Upregulated pathways in tumors with NEv2-like subtype

REACTOME_NUCLEAR_RECEPTOR_TRANSCRIPTION_PATHWAY

PID_RXR_VDR_PATHWAY

REACTOME_NR1H2_NR1H3_REGULATE_GENE_EXPRESSION_LINKED_TO_LIPOGENESIS

REACTOME_NR1H2_NR1H3_REGULATE_GENE_EXPRESSION_LINKED_TO_GLUCONEOGENESIS

REACTOME_NR1H2_NR1H3_REGULATE_GENE_EXPRESSION_LINKED_TO_TRIGLYCERIDE_LIPOLYSIS_IN_ADIPOSE

REACTOME_METABOLISM_OF_VITAMINS_AND_COFACTORS

HALLMARK_XENOBIOTIC_METABOLISM

REACTOME_METHYLATION

REACTOME_METAL_ION_SLC_TRANSPORTERS

REACTOME_SIGNALING_BY_RETINOIC_ACID

REACTOME_RA_BIOSYNTHESIS_PATHWAY

KEGG_RENIN_ANGIOTENSIN_SYSTEM

REACTOME_METABOLISM_OF_ANGIOTENSINOGEN_TO_ANGIOTENSINS

REACTOME_BETA_OXIDATION_OF_PRISTANOYL_COA

REACTOME_EXTRINSIC_PATHWAY_OF_FIBRIN_CLOT_FORMATION

REACTOME_SYNTHESIS_OF_BILE_ACIDS_AND_BILE_SALTS_VIA_27_HYDROXYCHOLESTEROL

REACTOME_ETHANOL_OXIDATION

REACTOME_ABACAVIR_TRANSPORT_AND_METABOLISM

REACTOME_PHASE_I_FUNCTIONALIZATION_OF_COMPOUNDS

REACTOME_PHASE1_FUNCTIONALIZATION_OF_COMPOUNDS

REACTOME_CYTOCHROME_P450_ARRANGED_BY_SUBSTRATE_TYPE

REACTOME_BIOLOGICAL_OXIDATIONS

BIOCARTA_NUCLEARRS_PATHWAY

REACTOME_PLASMA_LIPOPROTEIN_ASSEMBLY_REMODELING_AND_CLEARANCE

REACTOME_HEME_DEGRADATION

KEGG_COMPLEMENT_AND_COAGULATION_CASCADES

REACTOME_COMPLEMENT_CASCADE

BIOCARTA_LECTIN_PATHWAY

REACTOME_SYNTHESIS_OF_LEUKOTRIENES_LT_AND_EOXINS_EX

REACTOME_EICOSANOIDS

KEGG_ARACHIDONIC_ACID_METABOLISM

REACTOME_ARACHIDONIC_ACID_METABOLISM

REACTOME_PYRIMIDINE_CATABOLISM

REACTOME_ABACAVIR_TRANSMEMBRANE_TRANSPORT

BIOCARTA_MRP_PATHWAY

REACTOME_METABOLIC_DISORDERS_OF_BIOLOGICAL_OXIDATION_ENZYMES

REACTOME_ENDOGENOUS_STEROLS

REACTOME_TRANSPORT_OF_SMALL_MOLECULES

REACTOME_TRANSPORT_OF_BILE_SALTS_AND_ORGANIC_ACIDS_METAL_IONS_AND_AMINE_COMPOUNDS

KEGG_GLUTATHIONE_METABOLISM

REACTOME_GLUTATHIONE_CONJUGATION

REACTOME_NICOTINATE_METABOLISM

REACTOME_NICOTINAMIDE_SALVAGING

REACTOME_SYNTHESIS_OF_LIPOXINS_LX

KEGG_NICOTINATE_AND_NICOTINAMIDE_METABOLISM

REACTOME_METABOLISM_OF_WATER_SOLUBLE_VITAMINS_AND_COFACTORS

REACTOME_SYNTHESIS_OF_PROSTAGLANDINS_PG_AND_THROMBOXANES_TX

BIOCARTA_PPARA_PATHWAY

REACTOME_METABOLISM_OF_CARBOHYDRATES

HALLMARK_ADIPOGENESIS

REACTOME_HYALURONAN_UPTAKE_AND_DEGRADATION

REACTOME_HYALURONAN_METABOLISM

REACTOME_SIGNALING_BY_NUCLEAR_RECEPTORS

HALLMARK_ANDROGEN_RESPONSE

REACTOME_MOLYBDENUM_COFACTOR_BIOSYNTHESIS

REACTOME_ADRENOCEPTORS

Upregulated pathways in liver metastasis

REACTOME_MYOGENESIS

REACTOME_REGULATION_OF_COMMISSURAL_AXON_PATHFINDING_BY_SLIT_AND_ROBO

REACTOME_ACTIVATION_OF_RAC1

REACTOME_SIGNALING_BY_ROBO_RECEPTOR

REACTOME_SEMA3A_PAK_DEPENDENT_AXON_REPULSION

PID_NCADHERIN_PATHWAY

KEGG_GAP_JUNCTION

REACTOME_TP53_REGULATES_TRANSCRIPTION_OF_ADDITIONAL_CELL_CYCLE_GENES_WHOSE_EXACT_ROLE_IN_THE_P53_PATHWAY_REMAIN_UNCERTAIN

REACTOME_CA2_PATHWAY

PID_BETA_CATENIN_NUC_PATHWAY

REACTOME_NRAGE_SIGNALS_DEATH_THROUGH_JNK

REACTOME_SIGNALING_BY_RHO_GTPASES

PID_TELOMERASE_PATHWAY

PID_RHOA_PATHWAY

PID_NEPHRIN_NEPH1_PATHWAY

REACTOME_NTRK2_ACTIVATES_RAC1

REACTOME_ACTIVATED_NTRK2_SIGNALS_THROUGH_FYN

REACTOME_SIGNALING_BY_NTRK2_TRKB

REACTOME_L1CAM_INTERACTIONS

REACTOME_RECYCLING_PATHWAY_OF_L1

REACTOME_NRCAM_INTERACTIONS

REACTOME_NEUROFASCIN_INTERACTIONS

REACTOME_FACTORS_INVOLVED_IN_MEGAKARYOCYTE_DEVELOPMENT_AND_PLATELET_PRODUCTION

REACTOME_IONOTROPIC_ACTIVITY_OF_KAINATE_RECEPTORS

REACTOME_ACTIVATION_OF_KAINATE_RECEPTORS_UPON_GLUTAMATE_BINDING

REACTOME_COPI_INDEPENDENT_GOLGI_TO_ER_RETROGRADE_TRAFFIC

REACTOME_TRANSPORT_OF_CONNEXONS_TO_THE_PLASMA_MEMBRANE

REACTOME_SEALING_OF_THE_NUCLEAR_ENVELOPE_NE_BY_ESCRT_III

BIOCARTA_IRES_PATHWAY

REACTOME_RNA_POLYMERASE_II_TRANSCRIPTION

REACTOME_RECYCLING_OF_EIF2_GDP

REACTOME_MECP2_REGULATES_NEURONAL_RECEPTORS_AND_CHANNELS

REACTOME_POST_NMDA_RECEPTOR_ACTIVATION_EVENTS

REACTOME_CREB_PHOSPHORYLATION_THROUGH_THE_ACTIVATION_OF_RAS

REACTOME_ACTIVATION_OF_NMDA_RECEPTOR_UPON_GLUTAMATE_BINDING_AND_POSTSYNAPTIC_EVENTS

REACTOME_RAS_ACTIVATION_UPON_CA2_INFLUX_THROUGH_NMDA_RECEPTOR

REACTOME_CREB1_PHOSPHORYLATION_THROUGH_NMDA_RECEPTOR_MEDIATED_ACTIVATION_OF_RAS_SIGNALING

REACTOME_OPIOID_SIGNALLING

REACTOME_G_PROTEIN_MEDIATED_EVENTS

REACTOME_DAG_AND_IP3_SIGNALING

REACTOME_INTEGRATION_OF_PROVIRUS

REACTOME_EARLY_PHASE_OF_HIV_LIFE_CYCLE

BIOCARTA_CACAM_PATHWAY

PID_ER_NONGENOMIC_PATHWAY

KEGG_WNT_SIGNALING_PATHWAY

REACTOME_PTK6_REGULATES_RHO_GTPASES_RAS_GTPASE_AND_MAP_KINASES

BIOCARTA_DREAM_PATHWAY

BIOCARTA_TSP1_PATHWAY

BIOCARTA_ETS_PATHWAY

REACTOME_SIGNALING_BY_NTRKS

BIOCARTA_EPHA4_PATHWAY

REACTOME_ACTIVATION_OF_RAS_IN_B_CELLS

BIOCARTA_MEF2D_PATHWAY

BIOCARTA_CALCINEURIN_PATHWAY

BIOCARTA_GPCR_PATHWAY

BIOCARTA_INTEGRIN_PATHWAY

KEGG_MAPK_SIGNALING_PATHWAY

REACTOME_RAP1_SIGNALLING

KEGG_COLORECTAL_CANCER

PID_MAPK_TRK_PATHWAY

PID_TCR_RAS_PATHWAY

KEGG_NEUROTROPHIN_SIGNALING_PATHWAY

BIOCARTA_MAPK_PATHWAY

PID_RAS_PATHWAY

REACTOME_G_ALPHA1213_SIGNALLING_EVENTS

REACTOME_EFFECTS_OF_PIP2_HYDROLYSIS

REACTOME_G_PROTEIN_BETA_GAMMA_SIGNALLING

REACTOME_CD28_DEPENDENT_PI3K_AKT_SIGNALING

REACTOME_CD28_CO_STIMULATION

REACTOME_CD28_DEPENDENT_VAV1_PATHWAY

REACTOME_CD209_DC_SIGN_SIGNALING

PID_TCR_JNK_PATHWAY

BIOCARTA_TCR_PATHWAY

KEGG_T_CELL_RECEPTOR_SIGNALING_PATHWAY

BIOCARTA_BCR_PATHWAY

PID_IL2_1PATHWAY

PID_P38_ALPHA_BETA_PATHWAY

BIOCARTA_STATHMIN_PATHWAY

REACTOME_SUPPRESSION_OF_PHAGOSOMAL_MATURATION

REACTOME_RESPONSE_OF_MTB_TO_PHAGOCYTOSIS

REACTOME_PREVENTION_OF_PHAGOSOMAL_LYSOSOMAL_FUSION

PID_HDAC_CLASSII_PATHWAY

REACTOME_TRANSCRIPTIONAL_REGULATION_BY_RUNX1

REACTOME_SOS_MEDIATED_SIGNALLING

REACTOME_NOTCH2_INTRACELLULAR_DOMAIN_REGULATES_TRANSCRIPTION

REACTOME_RUNX1_REGULATES_GENES_INVOLVED_IN_MEGAKARYOCYTE_DIFFERENTIATION_AND_PLATELET_FUNCTION

REACTOME_P2Y_RECEPTORS

REACTOME_MECP2_REGULATES_TRANSCRIPTION_FACTORS

REACTOME_GP1B_IX_V_ACTIVATION_SIGNALLING

REACTOME_BICARBONATE_TRANSPORTERS

Description: In addition to previously known pathways upregulated in NE (cell cycle & proliferation, DNA damage repair & replication) or non-NE SCNCs (immune response, cell adhesion, metabolism) as indicated in Fig. 4e, we identified 100 co-clustered pathways regulating drug metabolism highly upregulated in specific tumors. We named these pathways "NEv2-like pathways" given a previous study showing a novel transcriptomic subtype characterized by upregulation of these drug metabolism pathways (Wooten DJ et al. 2019 Oct 31;15(10):e1007343.).Given that the NEv2-like tumors were more likely to be derived from liver metastases (9/11, 81.8%), we also evaluated pathways significantly upregulated (FDR < 1%) in liver biopsied tumors compared with other metastatic sites. Among the 90 pathways specifically upregulated in liver-derived tumors, none were overlapping with the NEv2-like pathways.

Abbreviations: SCNC: small cell neuroendocrine cancer; FDR: false discovery rate.
