# Supplementary material for: Heterogeneity of neuroendocrine transcriptional states in metastatic small cell lung cancers and patient-derived models
Source: Nat Commun. 2022 Apr 19;13:2023. doi: 10.1038/s41467-022-29517-9 (PMC9018864; doi:10.1038/s41467-022-29517-9)
Supplement: Supplementary file 3 — Description of Additional Supplementary Files [file 41467_2022_29517_MOESM3_ESM.pdf]

Description of Additional Supplementary Files

File Name: Supplementary Data 1
Description: Subtype-specific gene signatures
Subtype-specific gene signatures were derived from the top 500 contributors to PC1 (for SCNC-Y) and PC2 (for SCNC-A and SCNC-N) (see PCA plot in Fig S10a). The SCNC-Y gene signature is composed of the top 217 negative contributors to PC1. The SCNC-A gene signature is composed of the top 179 negative contributors to PC2. The SCNC-N gene signature is composed of the top 321 positive contributors to PC2. Abbreviations: PC: principal component; SCNC: small cell neuroendocrine cancers

File Name: Supplementary Data 2
Description: Enriched GO terms in SCNC subtypes
The GO enrichment analysis was performed for each subtype, using the subtype-specific gene lists. Abbreviations: FDR: false discovery rate; GO: gene ontology; SCNC: small cell neuroendocrine cancers

File Name: Supplementary Data 3
Description: Metabolic pathway enrichment analysis
Pathway ID: Metabolic pathways from KEGG, Hallmark, Reactome and biocarta; Pearson R: Pearson correlation between NE score and metabolic pathway ssGSEA enrichment score

File Name: Supplementary Data 4
Description: PDX gene expression data
