# Supplementary material for: Heterogeneity of neuroendocrine transcriptional states in metastatic small cell lung cancers and patient-derived models
Source: Nat Commun. 2022 Apr 19;13:2023. doi: 10.1038/s41467-022-29517-9 (PMC9018864; doi:10.1038/s41467-022-29517-9)
Supplement: Supplementary file 8 — Reporting Summary [file 41467_2022_29517_MOESM8_ESM.pdf]

nature portfolio

Corresponding author(s): Anish Thomas

Last updated by author(s): Jul 30, 2021

Reporting Summary

Nature Portfolio wishes to improve the reproducibility of the work that we publish. This form provides structure for consistency and transparency in reporting. For further information on Nature Portfolio policies, see our Editorial Policies and the Editorial Policy Checklist.

Statistics

For all statistical analyses, confirm that the following items are present in the figure legend, table legend, main text, or Methods section.

n/a	Confirmed	
☐	☒	The exact sample size (*n*) for each experimental group/condition, given as a discrete number and unit of measurement
☐	☒	A statement on whether measurements were taken from distinct samples or whether the same sample was measured repeatedly
☐	☒	The statistical test(s) used AND whether they are one- or two-sided<br>*Only common tests should be described solely by name; describe more complex techniques in the Methods section.*
☐	☒	A description of all covariates tested
☐	☒	A description of any assumptions or corrections, such as tests of normality and adjustment for multiple comparisons
☐	☒	A full description of the statistical parameters including central tendency (e.g. means) or other basic estimates (e.g. regression coefficient) AND variation (e.g. standard deviation) or associated estimates of uncertainty (e.g. confidence intervals)
☐	☒	For null hypothesis testing, the test statistic (e.g. *F*, *t*, *r*) with confidence intervals, effect sizes, degrees of freedom and *P* value noted<br>*Give P values as exact values whenever suitable.*
☒	☐	For Bayesian analysis, information on the choice of priors and Markov chain Monte Carlo settings
☒	☐	For hierarchical and complex designs, identification of the appropriate level for tests and full reporting of outcomes
☐	☒	Estimates of effect sizes (e.g. Cohen's *d*, Pearson's *r*), indicating how they were calculated

Our web collection on statistics for biologists contains articles on many of the points above.

Software and code

Policy information about availability of computer code

Data collection	No software or custom code were used
Data analysis	R studio version 1.3.1093 (R Foundation for Statistical Computing), GraphPad Prism version 8.1.2 (GraphPad Software), STATA software version 16.0 (Stata-Corp), and Qlucore Omics Explorer version 3.6(2.2) (Qlucore AB) were used to generate figures and statistical analyses.

For manuscripts utilizing custom algorithms or software that are central to the research but not yet described in published literature, software must be made available to editors and reviewers. We strongly encourage code deposition in a community repository (e.g. GitHub). See the Nature Portfolio guidelines for submitting code & software for further information.

Data

Policy information about availability of data

All manuscripts must include a data availability statement. This statement should provide the following information, where applicable:

- Accession codes, unique identifiers, or web links for publicly available datasets
- A description of any restrictions on data availability
- For clinical datasets or third party data, please ensure that the statement adheres to our policy

The raw data generated in this study (including phenotype, RNA-Seq and WES from human tumors) have been deposited in the database of Genotype and Phenotype (dbGaP) under accession code phs002541.v1.p1 (https://www.ncbi.nlm.nih.gov/projects/gap/cgi-bin/study.cgi?study_id=phs002541.v1.p1&phv=492899&phd=&pha=&pht=11494&phvf=&phdf=&phaf=&phtf=&dssp=1&consent=&temp=1). The individual-level data are available for download by authorized access only (https://dbgap.ncbi.nlm.nih.gov/aa/wga.cgi?login=&page=login). Please refer to the release notes for more details (https://ftp.ncbi.nlm.nih.gov/dbgap/studies/phs002541/phs002541.v1.p1/release_notes/Release_Notes.phs002541.SCLC_ChemoRefractory.v1.p1.MULTI.pdf).

Source data are provided with this paper. Additional publicly available datasets were used in this study, including early-stage SCLC tumors under accession code EGAS00001000925 (https://ega-archive.org/studies/EGAS00001000925), PDX (Supplementary Table S4 in the reference 28), CDX (https://zenodo.org/record/3574846#.YQLVuI77RPY) and cell line retrieved from CellMiner CDB: Small Cell Lung Cancer (https://discover.nci.nih.gov/SclcCellMinerCDB/).

Field-specific reporting

Please select the one below that is the best fit for your research. If you are not sure, read the appropriate sections before making your selection.

- [x] Life sciences
- [ ] Behavioural & social sciences
- [ ] Ecological, evolutionary & environmental sciences

For a reference copy of the document with all sections, see nature.com/documents/nr-reporting-summary-flat.pdf

Life sciences study design

All studies must disclose on these points even when the disclosure is negative.

Sample size	We evaluated 100 small cell neuroendocrine cancers (SCNC) acquired by biopsies from 72 patients, including 62 patients with small cell lung cancer (SCLC) and 10 patients with extrapulmonary small cell cancer (EPSCC). No sample size calculation was performed given that this is an observational study. The sample size may not ensure sufficient power to detect statistical significance in all the analyses.
Data exclusions	No data were excluded from the analysis
Replication	Not applicable given that this is an observational study.
Randomization	Not applicable given that this is an observational study.
Blinding	Not applicable given that this is an observational study.

Reporting for specific materials, systems and methods

We require information from authors about some types of materials, experimental systems and methods used in many studies. Here, indicate whether each material, system or method listed is relevant to your study. If you are not sure if a list item applies to your research, read the appropriate section before selecting a response.

Materials & experimental systems

n/a	Involved in the study	
☐	☒	Antibodies
☒	☐	Eukaryotic cell lines
☒	☐	Palaeontology and archaeology
☐	☒	Animals and other organisms
☐	☒	Human research participants
☐	☒	Clinical data
☒	☐	Dual use research of concern

Methods

n/a	Involved in the study	
☒	☐	ChIP-seq
☒	☐	Flow cytometry
☒	☐	MRI-based neuroimaging

Antibodies

Antibodies used	Immunohistochemistry (IHC) stains for synaptophysin (1:20; 790-4407, Roche), chromogranin (1:50; 760-2519, Roche) and INSM1 (1:1,000; sc-271408, Santa Cruz), were performed at National Institutes of Health (NIH), laboratory of Pathology, according to manufacturer's instruction. IHC staining for CD3 (pre-diluted; 790-4341, Roche) was done on multiple tissue sections of SCNC cases.
Validation	All antibodies are well-validated by manufacturers and are highly cited within the literature. Description from the manufacturer's websites:<br><br>- CONFIRM anti-CD3 (2GV6) Rabbit Monoclonal Primary Antibody (CONFIRM anti-CD3 (2GV6) antibody) is a rabbit monoclonal antibody (IgG) directed against the nonglycosylated epsilon chain of the human CD3 molecule.1 CONFIRM anti-CD3 (2GV6) antibody is intended for use to qualitatively identify T cells by light microscopy in sections of formalin-fixed, paraffin-embedded tissue on a VENTANA BenchMark IHC/ISH series of automated instruments. This product should be interpreted by a qualified pathologist in conjunction with histological examination, relevant clinical information and proper controls. This antibody is intended for in vitro diagnostic (IVD) use.<br><br>- Anti-Chromogranin A (LK2H10) Primary Antibody is directed against the chromogranin A protein found in the secretory granules of most neuroendocrine cells. This antibody exhibits a cytoplasmic staining pattern and may be used to aid in the identification of tumors of neuroendocrine origin. The antibody is intended for qualitative staining in sections of formalin-fixed, paraffin-embedded tissue. This product should be interpreted by a qualified pathologist in conjunction with histological examination, relevant clinical information and proper controls. This antibody is intended for in vitro diagnostic (IVD) use.

- Ventana Medical Systems' (Ventana) CONFIRM anti-Synaptophysin (SP11) Rabbit Monoclonal Primary Antibody is designed to qualitatively detect the presence of synaptophysin expressing cells via light microscopy in formalinfixed, paraffin-embedded tissue. Positive staining results may aid in the classification of neuroendocrine tumors. The clinical interpretation of any staining, or the absence of staining, must be complemented by morphological studies and evaluation of proper controls. Evaluation must be made by a qualified pathologist within the context of the patient's clinical history and other diagnostic tests. This antibody is intended for in vitro diagnostic (IVD) use.

- Anti-INSM1 Antibody (A-8) is a mouse monoclonal IgG1 κ INSM1 antibody, cited in 84 publications, provided at 200 µg/ml. Raised against amino acids 81-125 mapping near the N-terminus of INSM1 of human origin. Anti-INSM1 Antibody (A-8) is recommended for detection of INSM1 of mouse, rat and human origin by WB, IP, IF, IHC(P) and ELISA.

Animals and other organisms

Policy information about studies involving animals; ARRIVE guidelines recommended for reporting animal research

Laboratory animals

Eight-week-old male and female NSG mice (NOD.Cg-Prkdc scid Il2rg tm1Wjl/SzJ; # 005557, The Jackson Laboratory, Bar Harbor, ME) were implanted subcutaneously with fresh patient-needle biopsy supported with Matrigel (Corning). For each PDX, one mouse was used to start the expansion and then two mice at each passage. Consistent 7 x 7 mm3 is implanted from passage P1 to P3 while maintaining tumor stock at each passage. Mice were monitored daily, with caliper measurements and body weights recorded bi-weekly; caliper monitoring was performed 3 times per week if necessary, for close monitoring. The patient derived xenograft (PDX) model was well characterized for consistency and reliability in vivo and at the histopathological level for small cell lung cancer and similarity with patient histopathology of origin.

The PDX tumor stock are viably frozen to ensure early passage of the model are well preserved. For any study plan with the model, the SCLC-PDX is revived through tumor passage in increasing number of recipient mice; for quality control the histopathology of donor tumors is verified at every passage as well as the take up rate. Before implanting any preclinical study cohort, we ensure that take up rate is at 100% in 2 donor passages prior study cohort implant.

Mice were on 12h light/dark cycle. Temperature of the rooms was between 68-74F, and humidity was 30-70%.

Wild animals

No wild animals were used in this study

Field-collected samples

No field-collected samples were used in this study

Ethics oversight

Mouse handling and procedures were conducted under an approved Animal Study Protocol according to Frederick National Laboratory Animal Care and Use Committee guidelines.

Note that full information on the approval of the study protocol must also be provided in the manuscript.

Human research participants

Policy information about studies involving human research participants

Population characteristics

Full population characteristics are listed in Supplementary Tables 2.

Recruitment

Patients self-referred or were referred by their treating physicians.

Ethics oversight

NCI IRB approved.

Note that full information on the approval of the study protocol must also be provided in the manuscript.

Clinical data

Policy information about clinical studies

All manuscripts should comply with the ICMJE guidelines for publication of clinical research and a completed CONSORT checklist must be included with all submissions.

Clinical trial registration

NCT02487095, NCT02484404, NCT02146170

Study protocol

Full clinical protocols for trials described above are available upon request.

Data collection

We collected 100 small cell neuroendocrine cancer tumors acquired by biopsies from 72 patients, including 62 patients with small cell lung cancer and 10 patients with extrapulmonary small cell cancer. The tumors were collected at the National Cancer Institute and the University of Rochester Medical Center. These samples were collected between April 2016 and March 2020. The RNA and whole exome were centrally delivered to National Cancer Institute and sequenced there.

Outcomes

The primary and secondary outcomes of the clinical trial were related to anti-tumor efficacy of the combination, and are described in detail in the clinical trial reported previously (PMIDs: 31063862 and 33848478).
